# Smoking and salivary microbiota: a cross-sectional analysis of an Italian alpine population

**DOI:** 10.1038/s41598-023-42474-7

**Published:** 2023-11-02

**Authors:** Giacomo Antonello, Freida Blostein, Deesha Bhaumik, Elyse Davis, Martin Gögele, Roberto Melotti, Peter Pramstaller, Cristian Pattaro, Nicola Segata, Betsy Foxman, Christian Fuchsberger

**Affiliations:** 1 Institute for Biomedicine, Eurac Research - Affiliated Institute of the University of Lübeck, Bolzano, Italy; 2 Department of Cellular, Computational and Integrative Biology, University of Trento, Trento, Italy; 3 School of Public Health – Epidemiology, University of Michigan, Ann Arbor, MI USA

**Keywords:** Microbiology, Biomarkers, Health care, Risk factors

## Abstract

The oral microbiota plays an important role in the exogenous nitrate reduction pathway and is associated with heart and periodontal disease and cigarette smoking. We describe smoking-related changes in oral microbiota composition and resulting potential metabolic pathway changes that may explain smoking-related changes in disease risk. We analyzed health information and salivary microbiota composition among 1601 Cooperative Health Research in South Tyrol participants collected 2017–2018. Salivary microbiota taxa were assigned from amplicon sequences of the 16S-V4 rRNA and used to describe microbiota composition and predict metabolic pathways. Aerobic taxa relative abundance decreased with daily smoking intensity and increased with years since cessation, as did inferred nitrate reduction. Former smokers tended to be more similar to Never smokers than to Current smokers, especially those who had quit for longer than 5 years. Cigarette smoking has a consistent, generalizable association on oral microbiota composition and predicted metabolic pathways, some of which associate in a dose-dependent fashion. Smokers who quit for longer than 5 years tend to have salivary microbiota profiles comparable to never smokers.

## Introduction

Smoking is a risk factor for several complex, chronic diseases including but not limited to respiratory diseases^1^, periodontitis^2–4^, oropharyngeal cancers^5,6^ and cardiovascular diseases^7^. Recently, alterations to oral microbiota composition have been observed in cases of periodontitis^8–11^, squamous cells carcinoma^12^, cardiovascular diseases^13,14^ and in cigarette smokers^15–19^ (Supplementary File 1, Table 1). Therefore, it is possible that smoking related changes in the oral microbiota contribute to the etiology of one or more chronic health conditions. The oral microbiota performs several functions, including playing an important role in the exogenous nitrate reduction pathway and hence blood pressure regulation via nitric oxide (NO)^20–23^. Diets high in nitrate increase the presence of oral nitrate-reducing bacteria (NRB), the most prevalent of which are species in the *Neisseria*, *Prevotella* and *Actinomyces* genera^24^. when NRB are present, salivary nitrate reduction increases^23,25^. Whether tobacco consumption directly or indirectly alters the relative abundance of nitrate reducing bacteria remains to be explored; however, smoking was reported to inhibit uptake of blood-circulating nitrate into saliva^26^.

The salivary microbiota composition varies by smoking habits. A 2016 meta-analysis of 1204 USA citizens from two national cohorts found that compared to former or never smokers, smokers had a decreased relative abundance of Proteobacteria, an increase of Actinobacteria and a lower proportion of aerobic taxa after adjustment for age and sex^15^. A 2019 study set in New York city confirmed and extended those findings showing that, in contrast to former or never smokers (N = 86), the salivary microbiota of smokers (N = 86) showed higher abundance of genera *Stomatobaculum*, *Megasphaera, Veillonella, Leptotrichia, Campylobacter* and *Treponema,* and lower abundance of *Neisseria*, *Lautropia*, *Haemophilus*, *Capnocytophaga*^16^. Studies conducted in Saudi Arabia^17^, Asia^27,28^ and Europe^19^ reported comparable findings (Supplementary File 1, Figure 1). In a meta-analysis with 1204 Americans, Wu and colleagues uniquely found that the relative abundance of classes Betaproteobacteria, Gammaproteobacteria and Flavobacteriia was inversely correlated with the number of cigarettes smoked daily and directly correlated with the years since quitting smoking^15^. While associations between smoking status and salivary microbial composition have been previously characterized in Americans, no study has described associations of the salivary microbiota composition and metabolic potential with daily smoking intensity or years since quitting in a European population.

This study adds to our understanding of the associations of the salivary microbiota taxonomic and predicted metabolic functional composition with smoking status, intensity (grams/day) and history (years since cessation) in a large, novel, homogeneous Italian cohort aged 18–91: the Cooperative Health Research In South Tyrol (CHRIS)^29^ Microbiome study (CHRISMB). We hypothesized that we would observe results consistent with the literature and some novel insights attributable to the unique characteristics of CHRISMB and the large sample size. We additionally hypothesized that the nitrate reduction pathways could be less abundant in smokers, given the previous findings of decreases of taxa in the *Neisseria* and *Haemophilus* genera, which harbor several NRB species^30^.

## Results

### Characteristics of study population in relation to smoking

After exclusions (see “Methods” and Supplementary File 1, Tables 2 and 3 for details), CHRISMB consisted of 1601 individuals with an average age of 45 years (range 18–91) and had slightly more females (52.9%) than males. Most had 20 or more natural teeth (72.1%). Almost half (45%) were Current or Former smokers; cigarettes were the primary source of tobacco for all but 5 participants. Smokers were more frequently males and younger than Never or Former smokers (Table 1). Former smokers quit smoking 17.96 years, on average (Range 0–61; median 16). When stratified by age group, Current and Former smokers aged 41–60 years with higher lifetime exposure to smoke tended to have fewer teeth than smokers with a lower cumulative exposure (Supplementary File 1, Figure 2).

**Table 1:** Distribution of selected demographic descriptors in relation to smoking status in the Cooperative Health Research in South Tyrol Microbiome (CHRISMB) study.

	Never (N = 880)	Former (N = 395)	Current (N = 326)	CHRISMB (N = 1601)	X^2^ *p* value
Sex					2.7e−07
Male	356 (40.5%)	222 (56.2%)	173 (53.1%)	751 (46.9%)	
Female	524 (59.5%)	173 (43.8%)	153 (46.9%)	850 (53.1%)	
Age category (years)					3.6e−19
18–30	238 (27.0%)	41 (10.4%)	130 (39.9%)	409 (25.5%)	
31–40	139 (15.8%)	73 (18.5%)	57 (17.5%)	269 (16.8%)	
41–50	196 (22.3%)	75 (19.0%)	64 (19.6%)	335 (20.9%)	
51–60	144 (16.4%)	112 (28.4%)	51 (15.6%)	307 (19.2%)	
61–70	93 (10.6%)	57 (14.4%)	23 (7.1%)	173 (10.8%)	
71+	70 (8.0%)	37 (9.4%)	1 (0.3%)	108 (6.7%)	
N° teeth (self-reported)					0.07
0	50 (5.7%)	23 (5.8%)	16 (4.9%)	89 (5.6%)	
1–9	57 (6.5%)	41 (10.4%)	20 (6.1%)	118 (7.4%)	
10–19	117 (13.3%)	74 (18.7%)	48 (14.7%)	239 (14.9%)	
20+	656 (74.5%)	257 (65.1%)	242 (74.2%)	1155 (72.1%)	
Gums health (self-reported)					0.87
Excellent	45 (5.1%)	18 (4.6%)	16 (4.9%)	79 (4.9%)	
Very good	188 (21.4%)	79 (20.0%)	64 (19.6%)	331 (20.7%)	
Good	291 (33.1%)	124 (31.4%)	87 (26.7%)	502 (31.4%)	
Average	229 (26.0%)	84 (21.3%)	99 (30.4%)	412 (25.7%)	
Poor	47 (5.3%)	24 (6.1%)	22 (6.7%)	93 (5.8%)	
Very poor	6 (0.7%)	2 (0.5%)	3 (0.9%)	11 (0.7%)	
Missing	74 (8.4%)	64 (16.2%)	35 (10.7%)	173 (10.8%)	

Per-column percentages were also reported in brackets. The whole cohort is included under the “CHRISMB” column. Significance was calculated as X^2^ test for categorical variables. Non-available measures were reported as “Missing”.

Salivary microbiota DNA sequencing of selected samples consisted of almost 36 million reads, with a median read count per sample of 22,308 (interquartile range: 11,884, full range 5283–65,837). After filtering by prevalence and minimum detection (see “Methods”), the dataset included 627 ASVs assigned to 82 genera (Supplementary File 1, Table 4).

### Qualitative smoking habits are associated with compositional and functional profiles of salivary genera

**Figure 1:**
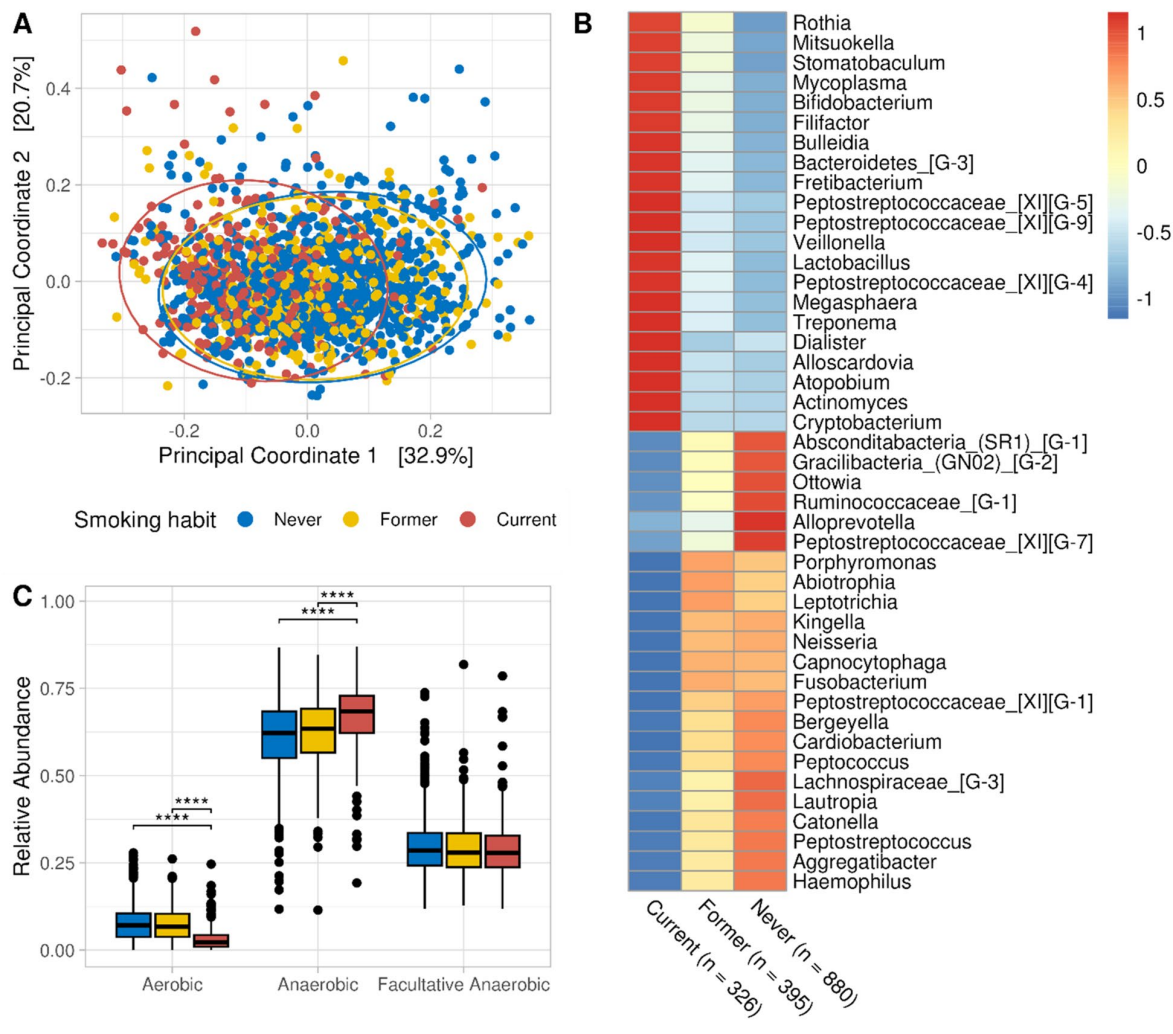
Association between qualitative smoking habits (Never, Former and Current) and the salivary microbiota in the CHRISMB cohort. (**A**) Principal Coordinate Analysis on the Bray–Curtis dissimilarity at genus level. Confidence areas (95%) were drawn as ellipses. Group separations were mild but significant (PERMANOVA R^2^ = 0.04, *p* = 0.001, beta-dispersity *p* = 0.104). Axes x and y were chosen as the principal components which explained most of the overall microbiota variability, which is shown in square brackets. (**B**) Heatmap of the 44 genera differentially abundant between Current and Never smokers. Each genus was transformed to relative abundance and Z-score scaled. Red and blue colors indicate a higher and lower mean abundance, respectively, while yellow colors indicate no difference. Genera reported in the figure were differentially abundant (Benjamini–Hochberg q-value < 0.05, false discovery rate (FDR) = 5%, ALDEx2 Holm q-value < 0.05) in at least 4 out of 5 differential abundance methods (DESeq2, LinDA, MaAsLin2, ALDEx2, ANCOM-BC), adjusting for age (categorical), sex (binary) and number of teeth (categorical). (**C**) Relative abundance of aerobes, anaerobes, and facultative anaerobes in relation to smoking status. Statistical significance was calculated with pairwise Wilcoxon test adjusting p-values (q-values) for a 5% FDR with the Benjamini–Hochberg method (**q < 0.05; ***q < 0.001, ****q < 0.0001).

The microbiota composition of CHRISMB at phylum level was dominated by Firmicutes, followed by Bacteroidetes Proteobacteria, Fusobacteria and Actinomycetes; while at Genus level it was dominated by *Prevotella, Streptococcus, Veillonella, Haemophilus, Neisseria* (Supplementary File 1, Figure 3). The salivary microbiota was significantly associated with smoking (Fig. 1A, PERMANOVA R^2^ = 0.04, *p* = 0.001, 2000 permutations) as well as sex, age group and number of teeth, considering the marginal effect of all variables together (Supplementary File 1, Table 5). Alpha diversity was not significantly associated with smoking status (Supplementary File 1, Figure 4). Principal coordinate analysis and differential abundance analysis together suggested that the salivary microbiota of Former smokers was highly similar to Never smokers. Consensus-based differential abundance analysis identified 44 genera that were significantly different between Current smokers and Never smokers after adjusting for age, sex, and number of teeth (Fig. 1B). To investigate sex-dependent associations, we repeated the same consensus differential abundance analysis separately by sex, again adjusting for age and number of teeth. Despite finding sex-specific differentially abundant genera, all were in the set of 44 differentially abundant genera of the model adjusted for sex, age group, and number of teeth (Supplementary File 1, Figure 5). We annotated genera based on their oxygen requirements from a manually curated table by Calgaro et al.^31^, and observed that the relative abundance of aerobic taxa decreased consistently in smokers (from a median of 7% to 3%), in favor of anaerobes (Fig. 1C).

### Several microbial genera associated with smoking habits are also associated with the grams of tobacco smoked daily

**Figure 2:**
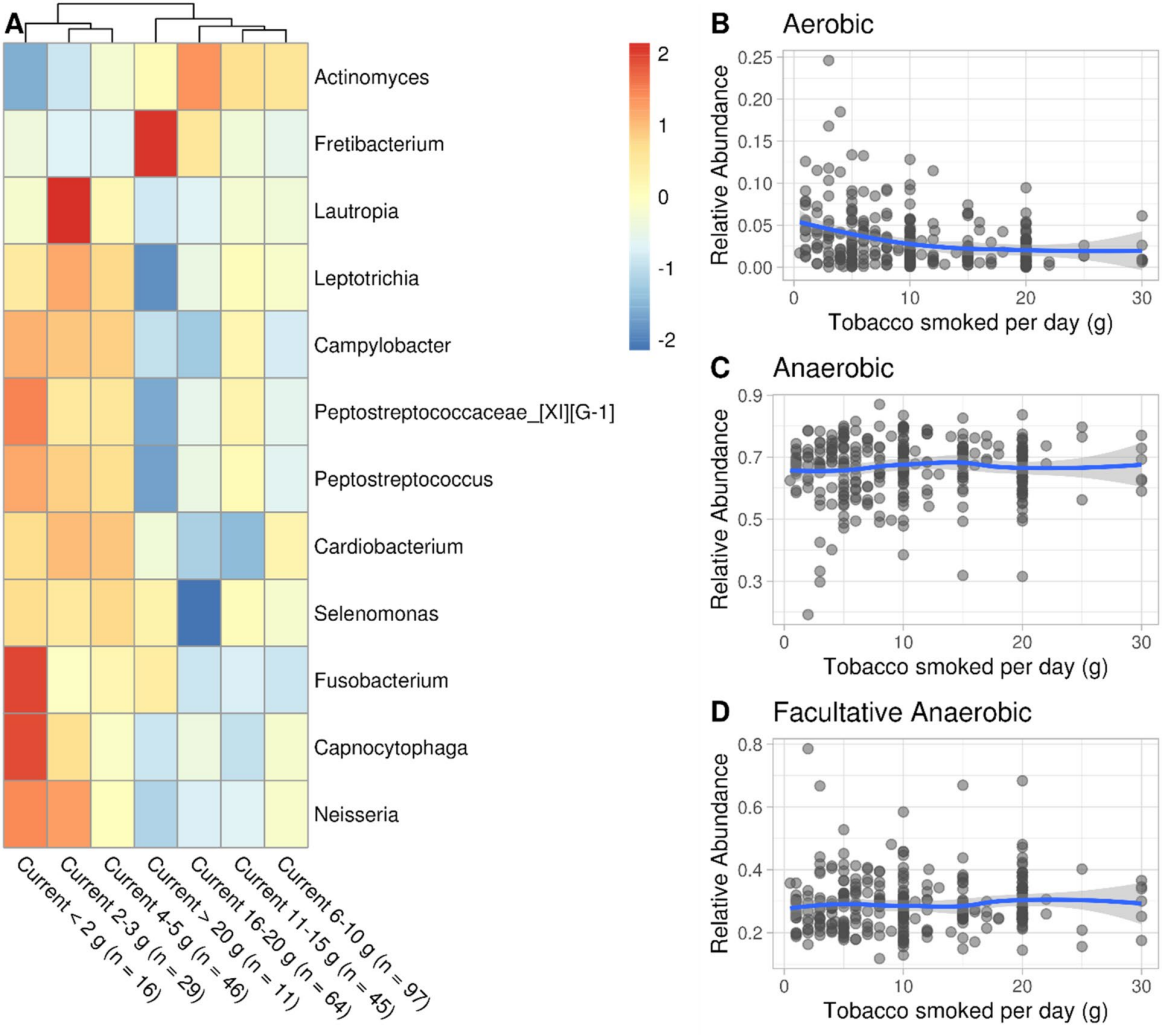
Smokers’ (n = 308) daily smoking intensity is associated with relative abundance shifts of several genera and a decrease of aerobic taxa relative abundance. (**A**) Heatmap of genera significantly affected by daily smoking intensity. Genera were transformed to relative abundance and Z-score scaled to highlight relative differences in mean abundance in relation to the smoking intensity. Significant genera (Benjamini–Hochberg q-value < 0.05, FDR = 5%) were obtained modeling each genus in response to daily smoking intensity as multiples of 5 g per day as a semi-continuous variable, adjusting for age (continuous), sex and number of teeth in the DESeq2 negative binomial generalized linear model framework. (**B**, **C**, **D**) Relative abundance of aerobes, anaerobes and facultative anaerobes, respectively, in relation to the grams of tobacco smoked daily.

**Figure 3:**
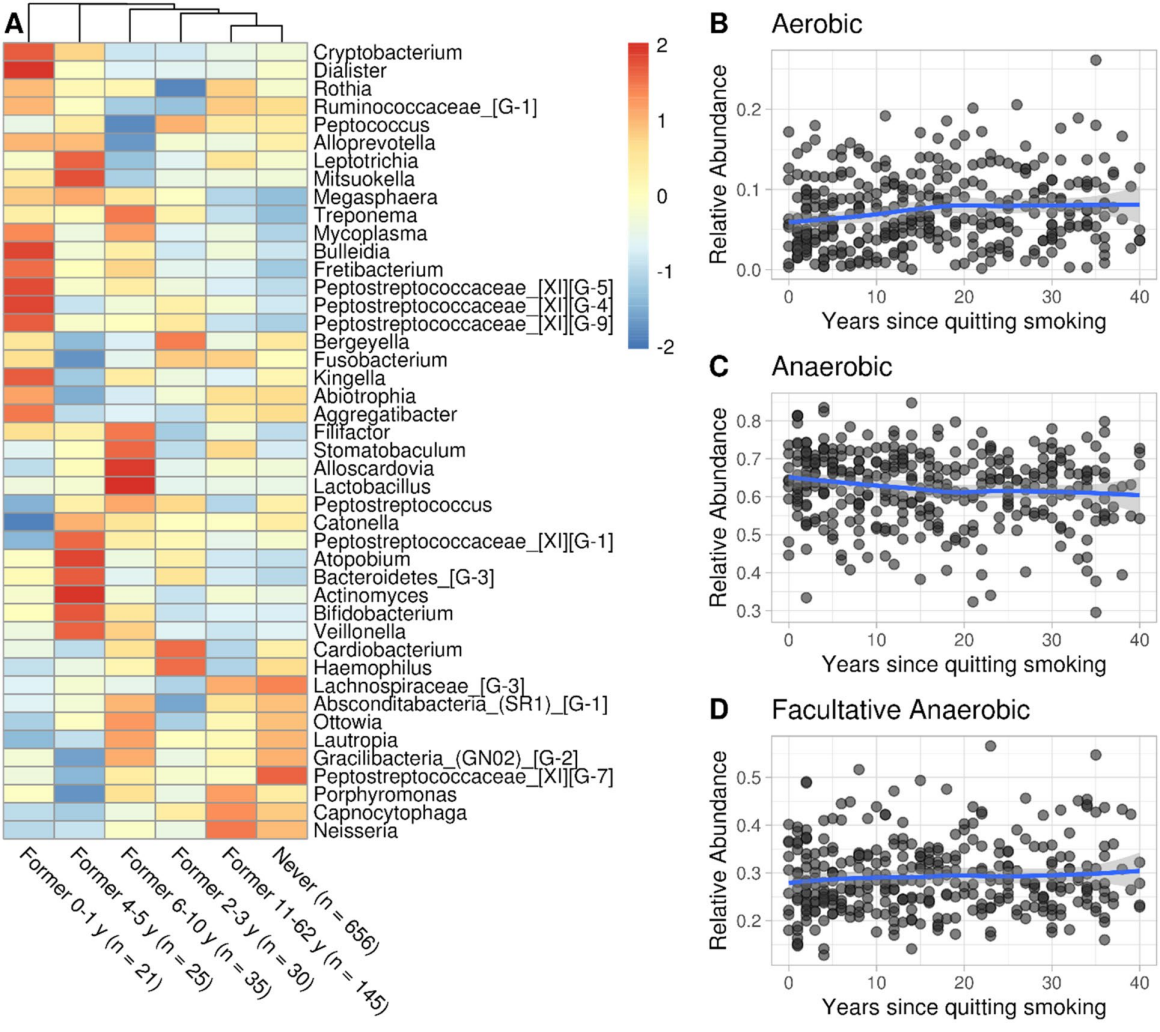
The salivary microbiota of individuals who quit smoking (n = 369) showed multiple-year perturbation and tends to resemble Never smokers’ profiles within 5 years. (**A**) Heatmap of the relationship between the years since quitting smoking and the mean relative abundance of genera previously found significantly associated with smoking (see Fig. 1). Taxa were transformed to relative abundance and scaled by row, to highlight differences in mean abundance in relation to bins of years since quitting to limit the low sample size of some categories. Complete linkage hierarchical clustering was used to cluster columns. Since Former smokers tend to be older and given the tendency of the elderly to lose teeth, we limited the visualization to people with 20 or more teeth. (**B**, **C**, **D**) Relative abundance of anaerobes, aerobes and facultative anaerobes in relation to years since quitting smoking.

We regressed each genus against daily smoking intensity as multiples of 5 g per day (see “Methods”). *Fretibacterium* was positively associated with increases in daily smoking intensity and 10 with decreases (Fig. 2A). Except for *Campylobacter* and *Selenomonas*, the remaining 9 genera were also differentially abundant comparing Current against Never smokers (Fig. 1B). Additionally, the effect sizes estimated in the daily smoking intensity regression were highly correlated with the estimates obtained comparing Current against Never smokers (Pearson $\rho$ = 0.87, Supplementary File 1, Figure 6), suggesting that some genera associated with smoking against non-smoking were additionally associated with daily smoking intensity. The complete linkage hierarchical clustering in the *pheatmap* function tended to cluster heavier smokers together, further suggesting a dose effect (Fig. 2A). The mean relative abundance and variance of aerobes significantly decreased at the increasing daily smoking intensity (linear regression $\beta (\frac{1}{grams/day})= 0.027$ , *p* value = $4.6\times 1{0}^{-4}$; Supplementary File 1, Tables 6, 7), adjusted for age as continuous variable, sex and number of teeth; Figs. 2C, 3D), with a plateau at more than 10 g (Fig. 2B). Conversely, the relative abundance of anaerobes and facultative anaerobes slightly increased.

### Salivary microbiota of Former smokers who quit 5 years or longer tended to resemble Never smokers’ profiles

We studied the association between salivary genera of former smokers and the years since smoking cessation using the same model framework as the daily intensity regression (Fig. 2), with 1 year scale, finding no statistically significant association. We visualized the mean relative abundance of genera associated with smoking (Fig. 1B) in the Former smokers’ group with 20 or more natural teeth, grouping them by bins of years since quitting. We limited the visualization to individuals with 20 or more teeth to minimize the effect of tooth loss on the microbiota of Former smokers, who tended to be older than Current and Never smokers. Looking at the complete linkage hierarchical clustering, we noticed a gradual increase of similarity of Never smokers to Former smokers who quit for more years, except for the “Former 2–3 y” group. (Fig. 3A). The relative abundance of aerobes mildly increased in the first 20 years since quitting ($\beta { }_{0 \le years \le 20}= 0.001$, *p* value $0.052$, adjusted for age, sex and number of teeth; Supplementary File 1, Tables 8, 9) (Fig. 3B).

### Predicted functional profiles associated with smoking highlighted a decrease of aerobic and nitrate reducing taxa

**Figure 4:**
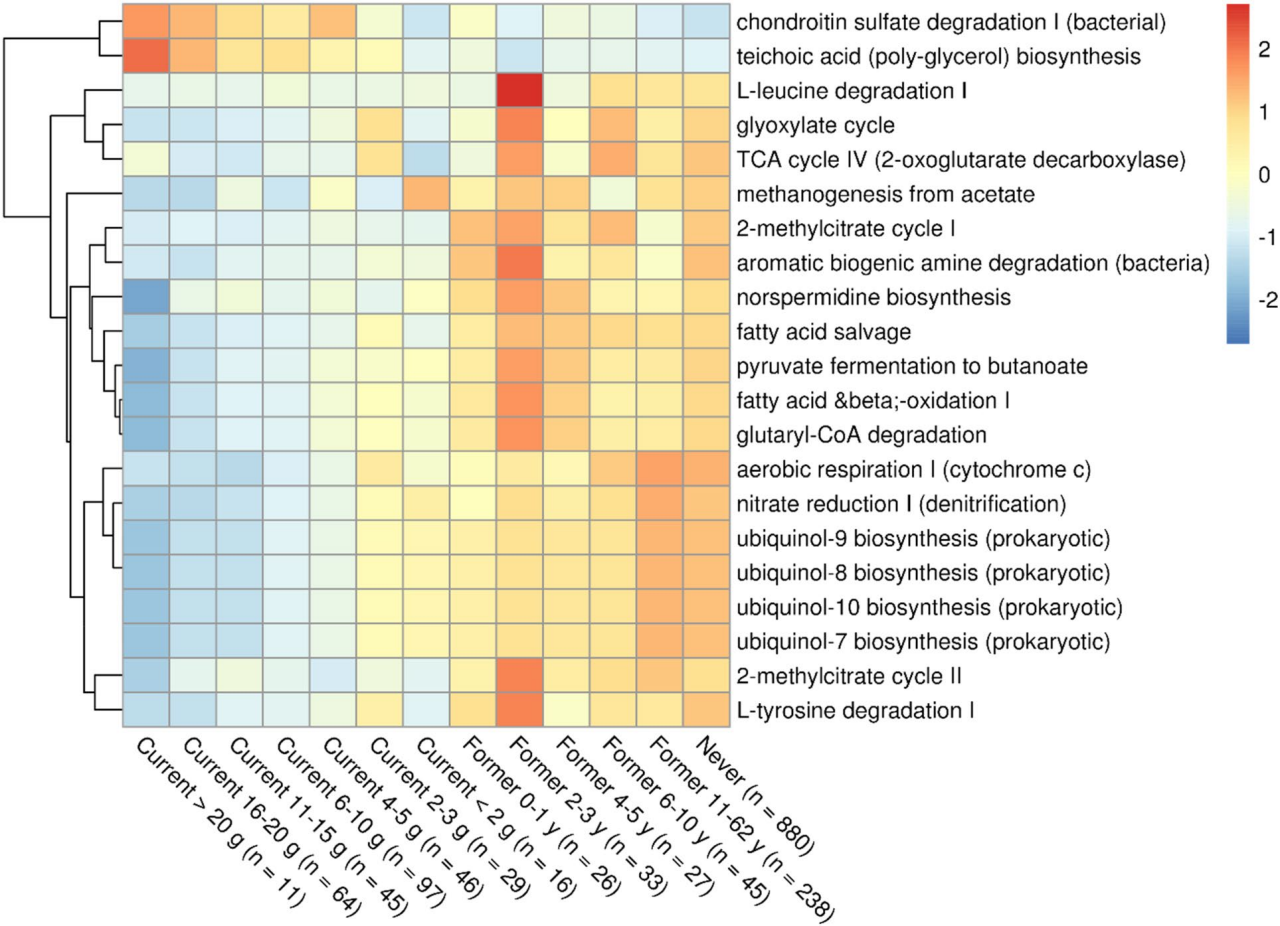
Microbial metabolic pathways inferred with PICRUSt2 that were differentially abundant in relation to smoking exposure, adjusting for age, sex and number of teeth. Heatmap of the 21 differentially abundant pathways in Current against Never smokers contrasts. Each pathway was transformed to relative abundance and Z-score scaled. Groups were ordered based on decreasing exposure to smoking, from heavier smokers to Former smokers who quit for the most years. As a reference for absence of exposure to smoking, never smokers were included in the rightmost column. Red and blue colors indicate a higher and lower mean abundance, respectively, while yellow colors indicate no difference. Differential abundance analysis was performed with a consensus-based approach of 5 differential abundance methods (DESeq2, LinDA, MaAsLin2, ALDEx2, ANCOM-BC), modeling each pathway against smoking status and adjusting for age (categorical), sex (binary) and number of teeth (categorical). Pathways reported in the figure were differentially abundant (Benjamini–Hochberg q-value < 0.05, False Discovery Rate = 5%, ALDEx2 Holm q-value < 0.05) in at least 4 methods with an absolute effect size larger than 0.5.

After predicting microbial pathway abundance with PICRUSt2, we identified pathways that were differentially abundant between Current and Never smokers using the same consensus method used for genus-level taxonomy. We identified 21 pathways, which we later visualized in relation to a gradient of smoking exposure, without clustering (Fig. 4). It should be noted that some of these were reconstructed from the same sets of predicted enzymes, therefore their correlation was 1 (e.g. Ubiquinol pathways). To avoid selection bias, we performed the analysis on all pathways regardless of their correlation and reported the correlation matrix of the significant ones in Supplementary File 1, Figure 7.

## Discussion

### Summary of study and main results

We investigated the associations between salivary microbial genera and predicted metabolic pathways and smoking status, daily smoking intensity and years since cessation in CHRISMB, a convenience sample of 1601 adult participants in the CHRIS study in South Tyrol, Italy^29^. We confirmed previous findings regarding salivary microbiota compositional differences by smoking behavior. Additionally, we demonstrated that aerobic taxa varied with the frequency and intensity of smoking exposure, and that the salivary microbiota of Former smokers is generally more similar to the salivary microbiota of Never smokers, especially of those who quit longer than 5 years. Several aerobic or oxygen-requiring predicted microbial pathways decreased in smokers. The nitrate reduction pathway was significantly lower in smokers than in non-smokers. The decreases in nitrate reduction pathways among current smokers and increases in these pathways among former smokers is consistent with previous reports of decreases in cardiovascular events among former smokers^32^. This suggests that oral microbiota functional changes with smoking may be an additional explanation for changes in cardiovascular risk with changes in smoking habits.

### Comparison with other studies

The relative abundance of salivary microbiota phyla of CHRISMB participants was comparable with the mean composition of a Japanese^27^ and Middle Eastern^17^: Firmicutes were the most abundant, followed by Bacteroidetes, Proteobacteria, Actinobacteria and Fusobacteria. Consistent with previous analyses in Americans of Caucasian, African and Hispanic ancestry^15,16^, and cohorts of middle^17^ and eastern Asian Ancestry^18,27^, Italian smokers had decreased abundance of *Neisseria*, *Lautropia*, *Haemophilus*, *Capnocytophaga,* and increased abundance of *Atopobium*, *Megasphaera* and *Veillonella* when compared to Never smokers (Fig. 1B). This suggests that cigarette smoking has a consistent and generalizable effect on the oral microbiota. We also identified 12 novel differentially abundant genera between Current and Never smokers: *Alloscardovia,* Bacteroidetes *Genus 3, Bulleidia*, *Cryptobacterium*, *Fretibacterium, Mitsuokella, Parvimonas,* Peptostreptococcaceae XI *Genus 9* and *Stomatobaculum* were increased, while Absconditabacteria (SR1) *Genus 1, Ottowia* and *Peptidiphaga* were decreased (Supplementary File 1, Figure 1). Further work is required to determine whether these changes are specific to this work.

### Salivary microbial genera composition and proportion of aerobes were strongly impacted by smoking

Of the 44 differentially abundant genera in smokers, compared to Never smokers, genera in the phylum Proteobacteria (N = 7) were decreased and Actinobacteria (N = 6) were increased among smokers. These two phyla harbor mostly aerobic and anaerobic taxa, respectively. Indeed, the proportion of aerobes was inversely proportional to the frequency and intensity of exposure to smoking (Figs. 1, 2, 3). We also predicted functional profiles based on our compositional data, observing an increase of Gram-positive associated pathways in smokers, in particular teichoic acid biosynthesis (Fig. 4), which we confirmed looking at the relative abundances of Gram staining of bacteria across smoking status (Supplementary File 1, Figure 8). Moreover, we observed a decrease in pathways associated with aerobes, such as nitrate reduction and ubiquinol synthesis, which is pivotal in the electron transport chain^33^, and a decrease of pathways that require oxygen and/or produce an excess reducing power, such as fatty acid oxidation. These findings support the hypothesis that smoking induces a hypoxic environment in the oral cavity.

A decreased abundance of the nitrate reduction pathway in smokers could be an effect of the decrease of genera *Neisseria, Haemophilus, Kingella*, which harbor several NRB. A decrease of NRB may have a detrimental effect on enterosalivary nitrate reduction^34^, which is a considerable source of blood nitrites for endogenous NO synthesis. Decreases in NO, which is a vasodilator^35^, might hinder gingival blood flow and increase stress over time, which could lead to higher chances of gingival recession and periodontal diseases^36^. Indeed, chondroitin sulfate degradation was increase in heavier smokers, which may be indicative of higher stress to the gingival connective tissue and increase the risk of periodontal diseases. NO deficiency has also been suggested as a risk factor for developing cardiovascular diseases^37–39^. Taken together, microbiota-derived NO depletion may increase the chance of developing periodontal and cardiovascular diseases in smokers, as recently reviewed^40^.

### Some genera are statistically associated with daily smoking intensity but not with the years since smoking cessation

In addition to examining quantitative differences by Current smoking status, we tested for differences in bacterial composition by daily intensity of tobacco exposure (g/day) (Fig. 2). Extending observations by Wu et al.^15^ at lower taxonomic level and higher resolution of exposure variables, genera belonging to classes Betaproteobacteria (*Lautropia, Neisseria)*, Gammaproteobacteria (*Cardiobacterium*) and Flavobacteriia (*Capnocytophaga*) were significantly decreased at increasing grams of tobacco smoked per day. Additionally, we found negative correlation with grams of tobacco smoked per day for genera in classes Clostridia (Peptostreptococcaceae Family XI—*Genus 1, Peptostreptococcus*), Epsilonproteobacteria (*Campylobacter*), Fusobacteriia (*Fusobacterium, Leptotrichia*) and Negativicutes (*Selenomonas*). Genera *Actinomyces* (class Actinobacteria) and *Fretibacterium* class Sinergistia) were significantly increased. The subsiding of smoking-related microbial taxa was in line with the observation of full recovery of cardiovascular health risk within 5 years since quitting^41^. It is possible that smoking induced oral microbiota alterations may last longer than 5 years (Fig. 3B,C,D), which would align with the subsiding of periodontal disease risks in smokers within 10 years^42^.

### Study limitations

Major limitations of this study include the cross-sectional design and the lack of a professional assessment of the number of decayed, missing and filled teeth and gum health. While we controlled for age, sex, and number of teeth as potential confounders in our models, residual confounding is still possible due to, for instance, medications usage, diet and alcohol intake. Furthermore, some subgroup strata were small, and structural non-positivity could exist. Bacterial metabolic pathways inference was based solely on salivary microbiota composition. While it is encouraging that our results regarding changes in salivary microbiota composition with smoking habits are consistent with those of previous studies conducted among very different populations, prospective studies are required to more directly address whether oral microbiota play a mediating role in the onset of smoking-related chronic diseases.

### Study strengths

Our analysis also has several strengths. The smoking questionnaire was detailed, allowing for high-resolution qualitative and quantitative characterization of smoking habits. We tested for a dose-dependent relationship between smoking and perturbation to the oral microbiota, supporting a causal relationship according to the Bradford Hill criteria^43^. This study cohort was particularly homogenous from the perspective of ethnicity, lifestyle and microbiota data generation, which should significantly limit confounding effects.

Our sample size was the largest to date to examine associations between smoking and the oral microbiota in a European population. While the salivary microbiota is a composite of multiple oral communities, saliva samples are easy to collect, making them ideal for large epidemiological cohorts and for future diagnostics and prognostics.

## Conclusions

Smoking is associated with changed in the salivary microbiota composition often in a dose-dependent fashion. The salivary microbiota of people who quit smoking longer than 5 years resembled Never smokers’ profiles. Irrespective of the phylogeny, aerobic taxa are the most sensitive to smoking exposure. Decreased microbial nitrate reduction pathway abundance in smokers may provide an additional explanation for the effect of smoking on cardiovascular and periodontal diseases risk, a hypothesis which should be tested in future studies.

## Materials and methods

### Study ethical approval, design, and data collection

The CHRIS study was approved by the local Ethical Committee within the South Tyrol healthcare on April 19, 2011, and registered with code 21.2011. The legal base for personal data handling and protection was the informed consent explained to and signed by each participant. The personal data protection warrant of CHRIS constantly ensures that all data are handled and protected in full compliance with the European Regulation (EU 2016/679) and Italian law (D.L.vo 196/2003).

The CHRIS study includes adults of both sexes aged 18 and older. Participants were recruited starting in 2011 with extensive outreach including advertisements, electronic and paper mail to cover most people residing in the Vinschgau/Val Venosta district (South Tyrol, Italy). On the day of visit, participants answered lifestyle, dietary, general health, and socio-economic status questionnaires^29^. The CHRIS Salivary microbiota (CHRISMB) project is a convenience sample of CHRIS participants recruited between January 2017 and February 2018.

#### Epidemiological data generation

We defined age as the difference between the examination date and the birth date, rounded to the closest integer, and categorized age into six groups as shown in Table 1. CHRISMB participants filled in an adapted version of the World Health Organization oral health survey^44^, from which we extracted information about the number of natural teeth in 4 ranges: 0, 1–9, 10–19 and 20 or more. We derived smoking variables from smoking questionnaires harmonized from the European Community Respiratory Health Survey III questionnaire^45^. We defined qualitative smoking habits—“Never”, “Former”, “Current with reduction”—Current (R), and “Current without reduction”—Current (NR)—according to Murgia et al.^46^. Former smokers were smokers who quit for longer than 1 month prior to the visit. Current (R) were individuals who reported being smokers at the day of examination but that reduced the daily smoking intensity at least 1 month prior to the visit. Since we did not observe differences in the microbiota composition of Current (R) and Current (NR) (Supplementary File 1, Figure 9), we decided to aggregate the two smoking groups. For completeness, included in the supplement is a description of the study population showing the separate characteristics of the Current and Former smoker groups (Supplementary File 1, Table 10). Cigarettes were the primary source of tobacco, except for 5 participants. To include all sources of tobacco as one variable of smoking intensity, we converted the number of cigarettes, cigars, and cigarillos into grams of tobacco equivalents, respectively 1, 5 and 3 g (g), and converted g/week to g/day as previously proposed^46,47^. We defined “smoking history” as the difference between the age of the participant to CHRIS and the reported age at which the participant quit smoking, rounded to the closest integer.

### Salivary microbiota data generation

#### Saliva sample collection and storage

CHRISMB participants were required to drink only water and fast from the night before. Additionally, they were required not to drink, eat or smoke within 1 h prior to the visit. During the visit, they provided 2–5 mL unstimulated saliva samples into Oragene OG-500 tubes. Within a few hours after the collection, samples were vortexed, split into 0.5 mL aliquots, and promptly stored at − 80 °C.

#### DNA extraction and sequencing

Salivary DNA extraction and sequencing were conducted by the University of Michigan microbiome core. DNA was extracted using the Eppendorf epMotion liquid handling system and Qiagen MagAttract PowerMicrobiome Kit protocol and quantified with the PicoGreen dsDNA Assay kit (Thermo Fisher Quant-iT, cat# P7589).We amplified the V4 hypervariable region of the 16S rRNA gene by polymerase chain reaction (PCR) using a dual indexing strategy^48^. PCR products were visualized using E-Gel 96 with 2% SYBR Safe DNA Gel Stain (Life Technologies cat# G7208-02). PCR products were then pooled and normalized using SequalPrep Normalization Plate Kit (Life Technologies, cat# A10510-01) following the manufacturer’s protocol for sequential elution.

The final pooled library consisted of equimolar amounts of each plate, normalized to the pooled plate at the lowest concentration. Sequencing libraries were prepared according to the Illumina MiSeq guidelines, adding phiX phage genome to ease diversity and quality control. Each of the 5 libraries contained 2 negative and 2 positive controls, respectively using water from the extraction step and commercially available DNA from communities of known composition from the PCR step (Zymo Research, cat# D6306). We sequenced reads on an Illumina MiSeq machine.

### Sequencing data processing

#### Sequencing data processing

We assessed the sequencing quality of the 69,286,448 obtained reads using “MultiQC” (v. 1.7) to visually determine read trimming length. We performed FASTQ read trimming, filtering, and taxonomic assignment with the “DADA2” package (v. 1.14)^49^ in R (v. 3.6.0)^50^. This method generates a high-resolution sequence table of amplicon sequence variants (ASVs), each differing by at least one nucleotide. We removed the first 20 and last 8 nucleotides to eliminate primer and barcode sequences and to ensure homogeneity of ASV calling across batches. After these steps, we submitted 59,331,563 reads to the *LearErrorRates* step, separately for each run, using 1 × 10^8^ bases as the learning rate parameter, which helps infer technical and real sequence differences. Then, we merged paired ends, resulting in 57,122,521 reads. Removal of chimeras using the consensus method resulted in an additional loss of 1.05% and 44,136,182 total reads used for taxonomic assignment. We assigned taxonomy from kingdom to genus level using the Bayesian classifier and the expanded Human Oral microbiome Database (eHOMD), while the species level was assigned using the 100% identity *addSpecies* strategy. To increase the likelihood of assignment at the species level, we enriched the eHOMD database with publicly available 16S rRNA FASTA sequences from known oral species in the genera *Lactobacillus*, *Streptococcus*, and *Prevotella* (Supplementary File 1, Table 11). We confirmed homogeneity across batches based on positive compositional profiles (Supplementary File 2).

#### Microbiota data preparation for analysis

We generated a phyloseq object starting from the counts table, taxonomic table and taxonomy tree using the Bioconductor package “phyloseq” (v. 1.42.0)^51^ and “ape” (v. 5.7). We retained only those taxa that were present with at least 10 reads in at least 1% of samples with the function *core* of the “microbiome” package (v. 1.20.0)^52^. We aggregated ASVs at the genus level with the *tax_glom* function in the GitHub package “speedyseq” (“mikemc/speedyseq”), a faster version of phyloseq for microbiome data manipulation.

### Samples availability for statistical analysis

Participants with missing data on smoking habits (N = 4), number of teeth (N = 44) and antibiotic usage within 3 months prior to the visit (N = 83) or who reported taking antibiotics within 3 months prior to saliva collection (N = 191) were excluded, leaving 1601 analytic samples. Additionally, we excluded 17 smokers from the “Regression of microbial genera against smoking intensity” due to missing or inconsistent grams of tobacco smoked per day and 1 participant who declared smoking 60 cigarettes per day, which was far beyond the range of the rest of the data (0.5–30). We further excluded 4 participants from the analysis “Regression of microbial genera against smoking history” due to inconsistent or missing answers.

### Statistical analysis

Unless reported otherwise, we performed all statistical analyses using R (v. 4.2.2) and RStudio Server (v. 2022.07.2).

#### Pairwise relationship between demographics

We tested the independence of smoking habits from age groups, sex, self-reported gum health and self-reported number of natural teeth using a χ^2^ test of independence with Yates’s correction for low-frequency groups. We considered traits with a p-value lower than 0.05 as statistically non-independent.

#### Beta diversity and dimensionality reduction visualization

We estimated between-sample microbiota dissimilarity transforming genera counts to relative abundance and calculating the Bray–Curtis dissimilarity with the *distance* function in “phyloseq”. We obtained eigenvectors with *ordinate* and visualized the two vectors explaining the most variance with *plot_ordination*, drawing 95% confidence interval ellipses with *stat_ellipse* in the “ggplot2” package (v. 3.4.0). We estimated the impact of smoking habits, number of teeth, sex and age group on the beta diversity using a permutational multivariate analysis of variance (PERMANOVA)^53^ with *adonis2* in the “vegan” package (v. 2.6-4), with 2000 permutations considering the marginal effect of all variables. We ensured even intraclass dispersion of smoking status groups (Never, Former and Current) using *betadisper* followed by *permutest*, with 2000 permutations (Supplementary File 1, Table 5).

#### Differential abundance analysis in relation to smoking

To study the association between each oral genus abundance and smoking, we performed differential abundance analysis comparing Current with Never smokers adjusting for age group, sex and number of teeth. We performed a consensus based differential abundance analysis, as advised by Nearing et al.^54^, using 5 different methods having: DESeq2 (v. 1.38.2)^55^, LinDA (v. 1.1)^56^, MaAsLin2 (v. 1.12)^57^, ALDEx2 (v. 1.30)^58^ and ANCOMBC (v. 1.6.4)^59^.

We defined significant differentially abundant genera if Benjamini–Hochberg (BH) corrected q-values were below 0.05 in at least 4 out of 5 methods with a false discovery rate (FDR) = 5%^60^. We used Holm multiple testing correction in ALDEx2 as it was the only method implemented in its generalized linear model (GLM) framework.

#### Regression of microbial genera against smoking intensity

To study the compositional changes of microbial genera in response to the grams of tobacco smoked per day, we modeled each genus in against the grams of tobacco per day as a continuous variable in a Negative binomial GLM (DESeq2). We binned the daily tobacco smoked into multiples of 5 g as those were the most frequent answers (Supplementary File 1, Figure 10). We considered genera as significant when BH-corrected q-values were lower than 0.05 with FDR = 5%.

#### Regression of microbial genera against smoking history

To study the compositional changes of microbial genera in response to smoking history, we modeled each genus in response to years since smoking cessation as a continuous variable, at 1-year interval in a Negative binomial GLM (DESeq2). We considered genera as significant when BH-corrected q-values were lower than 0.05 with FDR = 5%.

#### Insights into the functional potential of the salivary microbiota

We inferred the functional potential of the oral microbiota at the ASV level using *picrust2_pipeline.py* with default parameters implemented in PICRUSt2 (v. 2.5)^61^. We investigated differential abundant pathways with the same strategy used for genera differential abundance. We considered pathways as significant if the absolute effect size was above 0.5 and the q-value below 0.05 in at least 4 methods. To further confirm the impact of smoking in relation to the proportion of aerobic taxa, we mapped each genus to a table of curated annotations of three oxygen metabolism classes: aerobic, anaerobic and facultative anaerobic^31^. We visualized the relative abundance of aerobes, anaerobes and facultative anaerobes in each sample with respect to smoking status with pairwise Wilcoxon tests, correcting p-values with BH (FDR = 5%).

## Data and analysis scripts availability

CHRIS and CHRISMB data can be requested from https://chrisportal.eurac.edu/, upon approval of the researcher’s proposal by the CHRIS data access committee. Analysis scripts are freely accessible at https://github.com/g-antonello/CHRISMB-smoking-epidemiology.

### Supplementary Information

Supplementary Information 1.

Supplementary Information 2.

## Abbreviations

ASV: Amplicon sequence variant
BH: Benjamini–Hochberg
CHRIS: Cooperative Health Research in South Tyrol
CHRISMB: CHRIS microbiome project
eHOMD: Expanded Human Oral Microbiome Database
FDR: False discovery rate
g: Gram/grams
GLM: Generalized linear model
NO: Nitric oxide
NRB: Nitrate-reducing bacteria
PCR: Polymerase chain reaction
