# Supplementary material for: Smoking and salivary microbiota: a cross-sectional analysis of an Italian alpine population
Source: Sci Rep. 2023 Nov 2;13:18904. doi: 10.1038/s41598-023-42474-7 (PMC10622503; doi:10.1038/s41598-023-42474-7)
Supplement: Supplementary file 1 — Supplementary Information 1. [file 41598_2023_42474_MOESM1_ESM.pdf]

Smoking and salivary microbiota, a cross-sectional analysis of an Italian alpine population

Supplementary Figures and Tables

Giacomo Antonello, Freida Blostein, Deesha Bhaumik, Elyse Davis, Martin Gögele, Roberto Melotti, Peter Pramstaller, Cristian Pattaro, Nicola Segata, Betsy Foxman, Christian Fuchsberger

2023-08-20

Contents

Phyloseq Object used for the analyses (ASV level) **1**

Supplementary Figures **2**

Supplementary Figure 1 - Differentially Abundant genera in comparable literature 2

Supplementary Figure 2 - Tooth loss vs Age and Smoking 3

Supplementary Figure 3 - Barplots of Phylum and Genus level salivary microbiota composition in the CHRISMB population 4

Supplementary Figure 4 - Alpha diversity and richness in relation to smoking status 5

Supplementary Figure 5 - Sex specific differentially abundant genera with Smoking 7

Supplementary Figure 6 - Correlations log2 Fold Change values from Differential abundance and regression on daily smoking intensity 8

Supplementary Figure 7 - Correlation of differentially abundant pathways 9

Supplementary Figure 8 - Gram staining relative abundances vs Smoking habits 10

Supplementary Figure 9 - Current (NR) are indistinguishable from Current (R) 11

Supplementary Figure 10 - Histogram of grams per day in current smokers 12

Supplementary Tables **13**

Supplementary Table 1 - Literature overview, table of ethnicities and sample size of studies 13

Supplementary Table 2 - Available samples after each exclusion step 13

Supplementary Table 3 - No statistical differences between excluded and included participants for the study 14

Supplementary Table 4 - Per taxonomic level microbiota statistics 15

Supplementary Table 5 - PERMANOVA table of variables included in the models (marginal effect) 15

Supplementary Table 6 - Linear model aerobes against smoking intensity per day 16

Supplementary Table 7 - Linear model anaerobes against smoking intensity per day 16
Supplementary Table 8 - Linear model aerobes against years since smoking cessation 16
Supplementary Table 9 - Linear model anaerobes against years since smoking cessation 17
Supplementary Table 10 - Table 1 with Current smokers split into reducers and non-reducers . . . 17
Supplementary Table 11 - eHOMD database expansion, list of genomes accession numbers per species 18

Phyloseq Object used for the analyses (ASV level)

Unfiltered Phyloseq Object (Starting point, never used in the analyses)

```
phyloseq-class experiment-level object
otu_table()   OTU Table:         [ 11860 taxa and 1923 samples ]:
sample_data() Sample Data:       [ 1923 samples by 1505 sample variables ]:
tax_table()   Taxonomy Table:    [ 11860 taxa by 8 taxonomic ranks ]:
phy_tree()    Phylogenetic Tree: [ 11860 tips and 11858 internal nodes ]:
refseq()      DNAStringSet:      [ 11860 reference sequences ]
taxa are rows
```

Filtered Taxa and samples. This was the phyloseq used for the actual anlyses For further details, see Suppl. Tables 2 and 4

```
phyloseq-class experiment-level object
otu_table()   OTU Table:         [ 622 taxa and 1601 samples ]:
sample_data() Sample Data:       [ 1601 samples by 1505 sample variables ]:
tax_table()   Taxonomy Table:    [ 622 taxa by 8 taxonomic ranks ]:
phy_tree()    Phylogenetic Tree: [ 622 tips and 621 internal nodes ]:
refseq()      DNAStringSet:      [ 622 reference sequences ]
taxa are rows
```

Supplementary Figures

Supplementary Figure 1 - Differentially Abundant genera in comparable literature

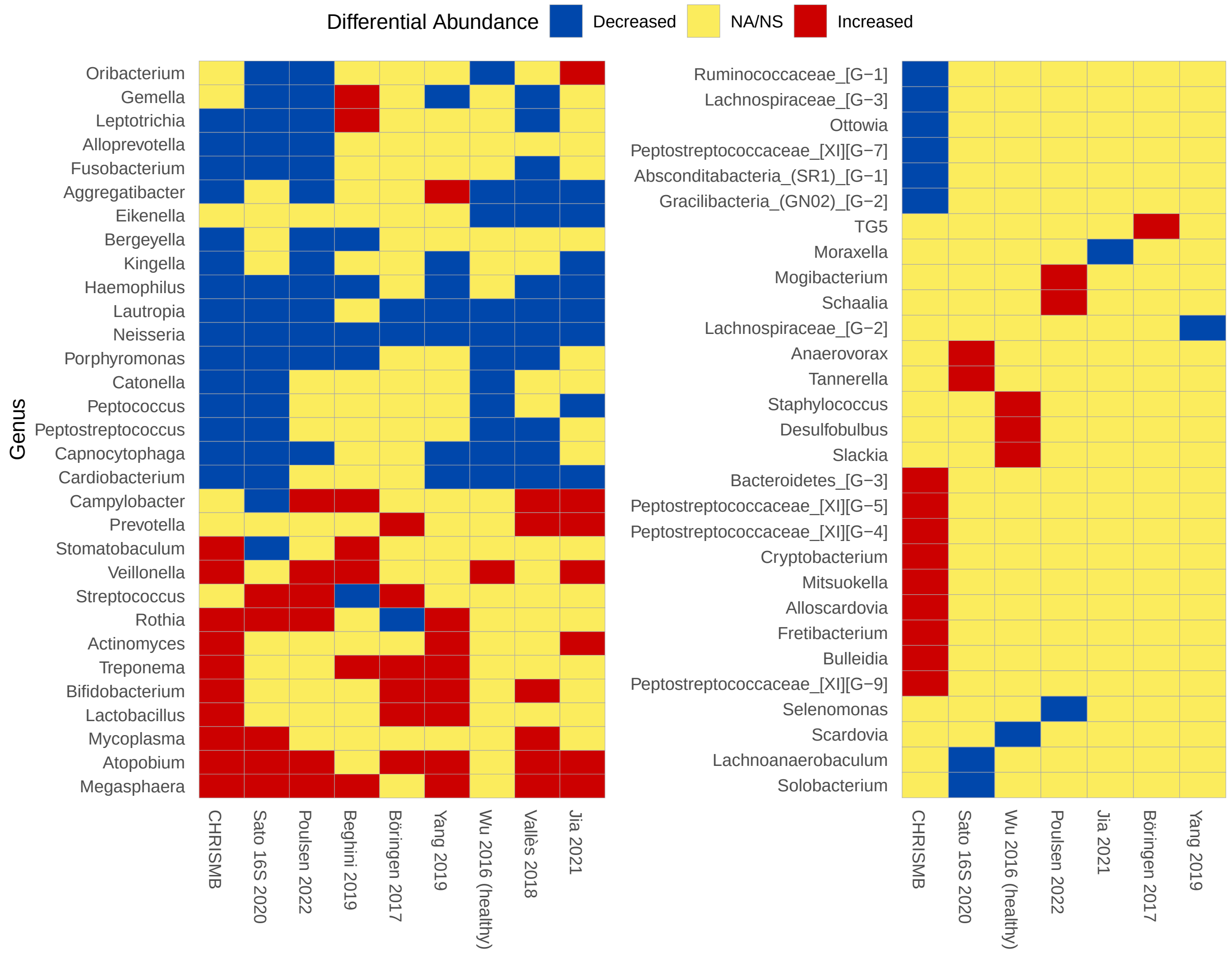

Supplementary Figure 1. Heatmap of differentially abundant genera in smokers compared to non-smokers both in the literature and in the current study (CHRISMB). Genera were manually annotated based on publications with comparable setup: cross-sectional studies on a healthy population, and 16S amplicon microbiota data. **Red** color indicates increased abundance in smokers, **Blue** means decreased abundance in smokers. Yellow means not significant (NS) or not mentioned (NA) in the text nor in the supplementary materials. **(Left)** Commonly reported genera (present in at least 3 studies). **(Right)** Rarely reported genera (present in only one study).

Supplementary Figure 2 - Tooth loss vs Age and Smoking

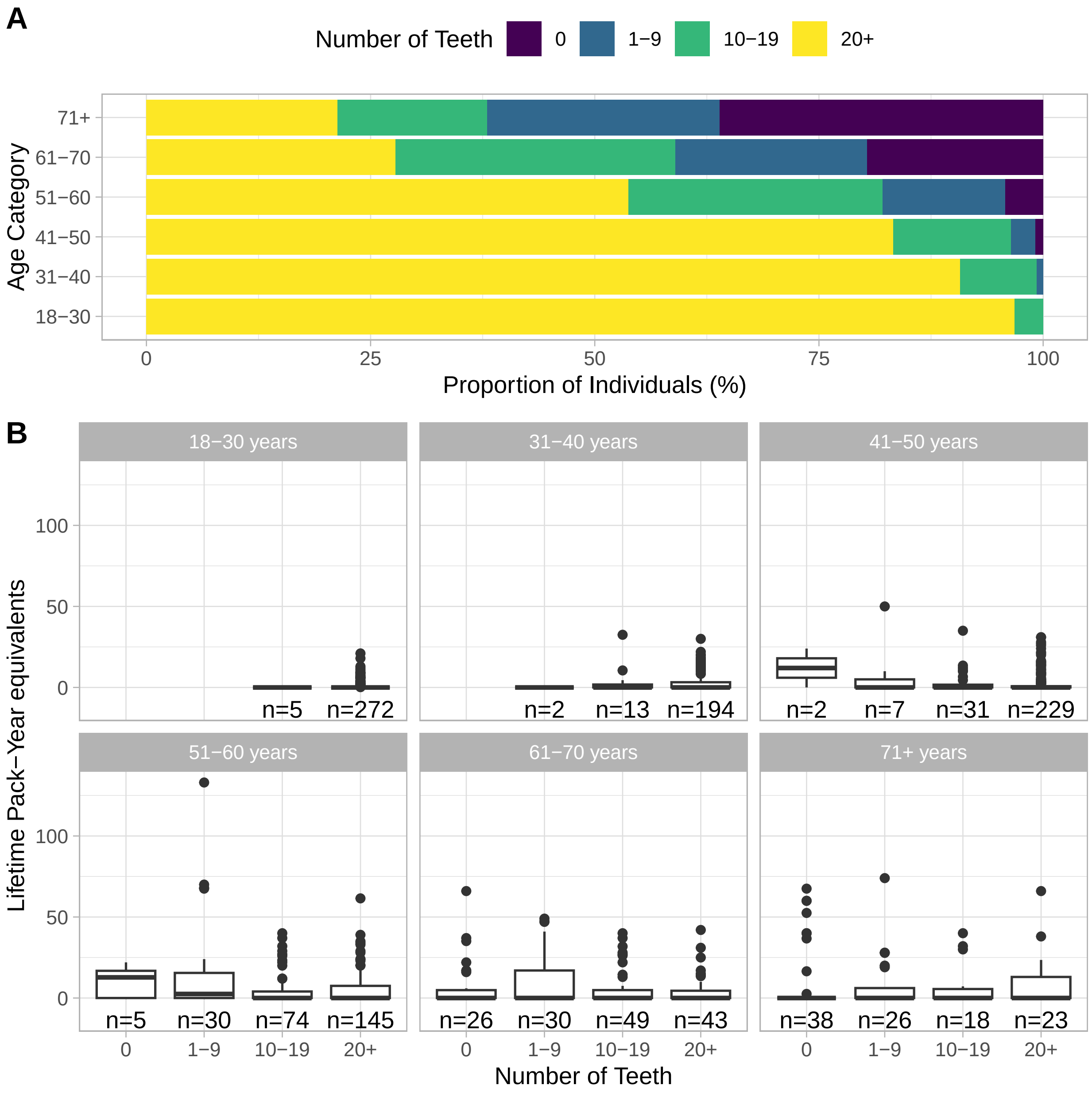

Supplementary Figure 2: Non-independence of number of teeth from age groups and smoking (A) Relationship between age and number of teeth. Numbers are reported as proportions for each age category. (B) Relationship between lifetime exposure to smoke (lifetime pack-years) and the number of teeth in only current smokers. Individuals were further split into age groups to control for age-related tooth loss. Lifetime pack years, a proxy of a lifetime exposure to the tobacco smoked, were calculated in Former and Current smokers as follows:

$$Lifetime\ Pack\text{-}Year\ equivalents = \frac{\frac{cigarettes}{day}}{20} \times 365\ days \times years\ smoked$$

Supplementary Figure 3 - Barplots of Phylum and Genus level salivary microbiota composition in the CHRISMB population

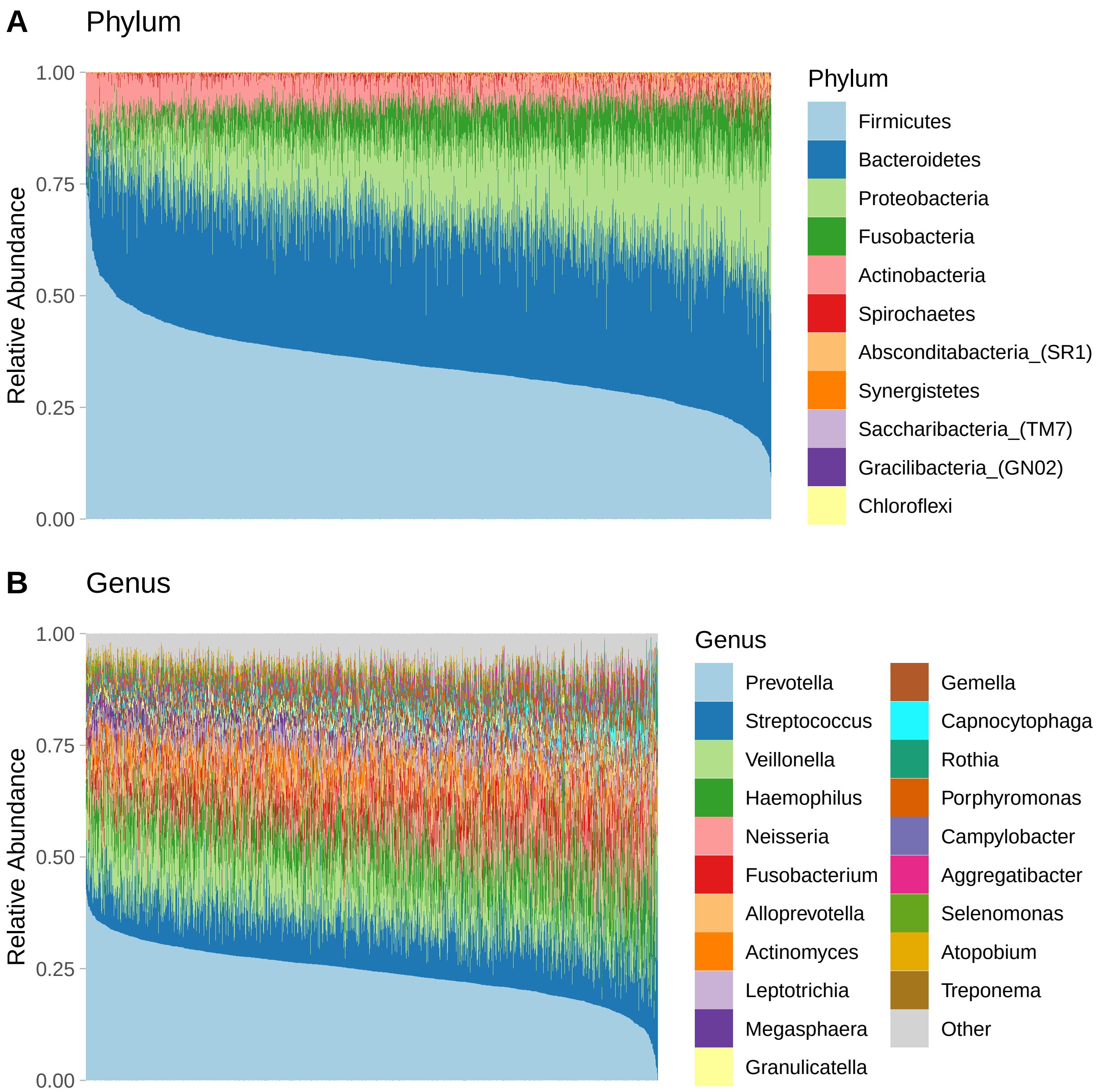

Supplementary Figure 3. Barplots of relative abundance composition of CHRISMB samples included in this study (N = 1601) Each bar is a sample. Samples were sorted from left to right based on the decreasing relative abundance of the most abundant taxa overall (A) Phylum level, samples were sorted by decreasing abundance of Firmicutes; (B) Genus level, samples were sorted by decreasing abundance of Prevotella.

Supplementary Figure 4 - Alpha diversity and richness in relation to smoking status

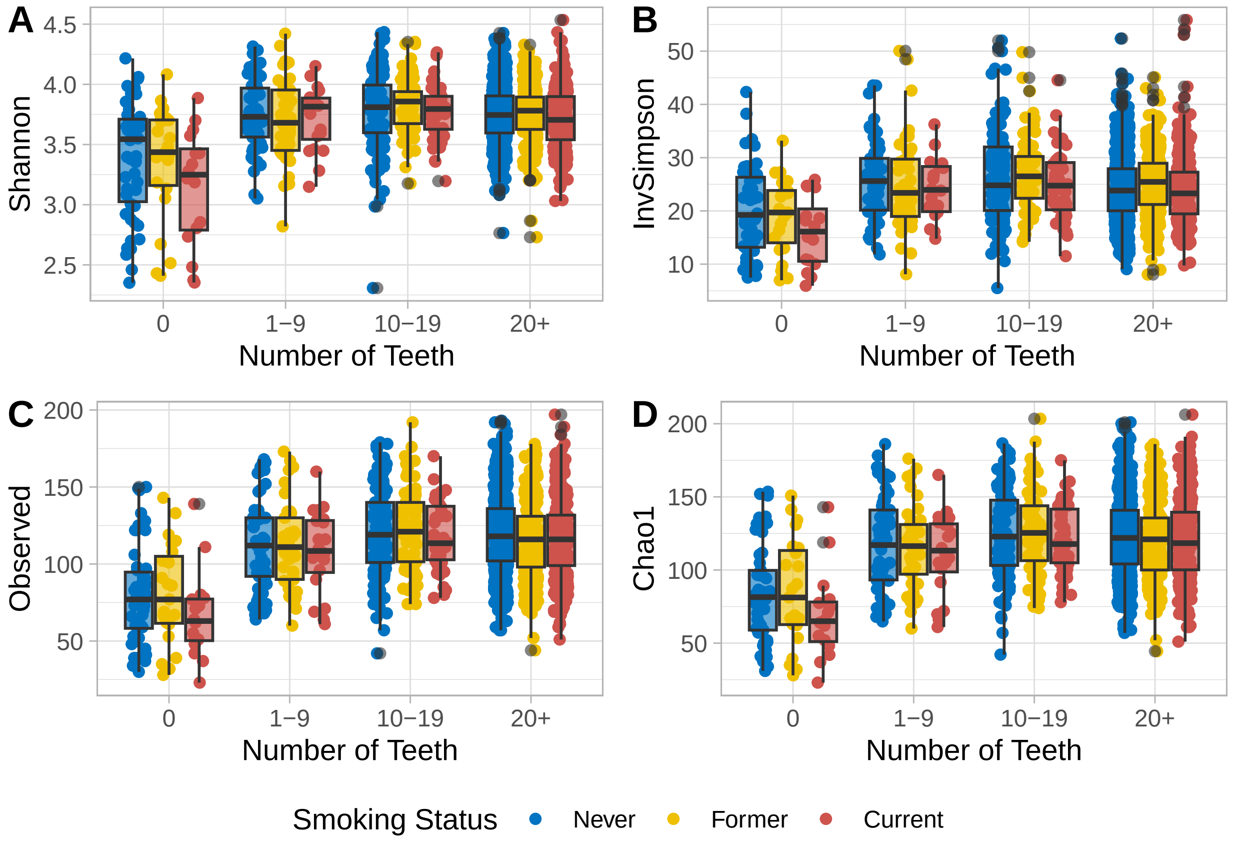

Supplementary Figure 4. Alpha diversity and richness estimates in relation to smoking (colours) subdivided by the number of teeth (x axis) (A) Shannon diversity; (B) Inverse Simpson diversity; (C) Observed number of taxa; (D) Chao 1 Richness. Estimates were calculated on samples rarefied to 5000 counts per sample.

Table 1: Shannon Diversity/Richness metric in relation to other variables considered in the study. Significance was estimated with the ‘stats::lm‘ linear regression function modeling each alpha metric against the following variables in the model: age, sex, Number of Teeth, Smoking Status. No, transformation was applied to any variable considered

term	estimate	std.error	statistic	p.value
Intercept	3.24	0.05	67.79	<2e-16
Smoking Status: Former	0.00	0.02	-0.04	0.966
Smoking Status: Current	-0.03	0.02	-1.82	0.068
Number of Teeth: 1-9	0.40	0.04	10.65	<2e-16
Number of Teeth: 10-19	0.49	0.03	14.22	<2e-16
Number of Teeth: 20+	0.46	0.03	13.77	<2e-16
Age	0.00	0.00	3.24	0.001
Sex: Female	-0.02	0.01	-1.19	0.233

Table 2: InvSimpson Diversity/Richness metric in relation to other variables considered in the study. Significance was estimated with the ‘stats::lm‘ linear regression function modeling each alpha metric against the following variables in the model: age, sex, Number of Teeth, Smoking Status. No, transformation was applied to any variable considered

term	estimate	std.error	statistic	p.value
Intercept	15.84	1.20	13.22	<2e-16
Smoking Status: Former	0.30	0.42	0.73	0.46
Smoking Status: Current	-0.45	0.45	-1.00	0.32
Number of Teeth: 1-9	6.36	0.95	6.70	3e-11
Number of Teeth: 10-19	7.77	0.86	9.06	<2e-16
Number of Teeth: 20+	6.91	0.84	8.24	3e-16
Age	0.05	0.01	4.15	3e-05
Sex: Female	-0.70	0.34	-2.04	0.04

Table 3: Observed Diversity/Richness metric in relation to other variables considered in the study. Significance was estimated with the ‘stats::lm‘ linear regression function modeling each alpha metric against the following variables in the model: age, sex, Number of Teeth, Smoking Status. No, transformation was applied to any variable considered

term	estimate	std.error	statistic	p.value
Intercept	76.87	4.65	16.55	<2e-16
Smoking Status: Former	-1.64	1.61	-1.01	0.31
Smoking Status: Current	-3.25	1.73	-1.89	0.06
Number of Teeth: 1-9	34.38	3.68	9.34	<2e-16
Number of Teeth: 10-19	43.55	3.33	13.10	<2e-16
Number of Teeth: 20+	41.05	3.25	12.63	<2e-16
Age	0.02	0.05	0.44	0.66
Sex: Female	0.33	1.32	0.25	0.80

Table 4: Chao1 Diversity/Richness metric in relation to other variables considered in the study. Significance was estimated with the ‘stats::lm‘ linear regression function modeling each alpha metric against the following variables in the model: age, sex, Number of Teeth, Smoking Status. No, transformation was applied to any variable considered

term	estimate	std.error	statistic	p.value
Intercept	81.42	4.94	16.49	<2e-16
Smoking Status: Former	-1.70	1.72	-0.99	0.32
Smoking Status: Current	-3.41	1.83	-1.86	0.06
Number of Teeth: 1-9	34.74	3.91	8.88	<2e-16
Number of Teeth: 10-19	43.90	3.53	12.42	<2e-16
Number of Teeth: 20+	41.12	3.45	11.90	<2e-16
Age	0.00	0.05	0.08	0.94
Sex: Female	0.40	1.41	0.29	0.77

Supplementary Figure 5 - Sex specific differentially abundant genera with Smoking

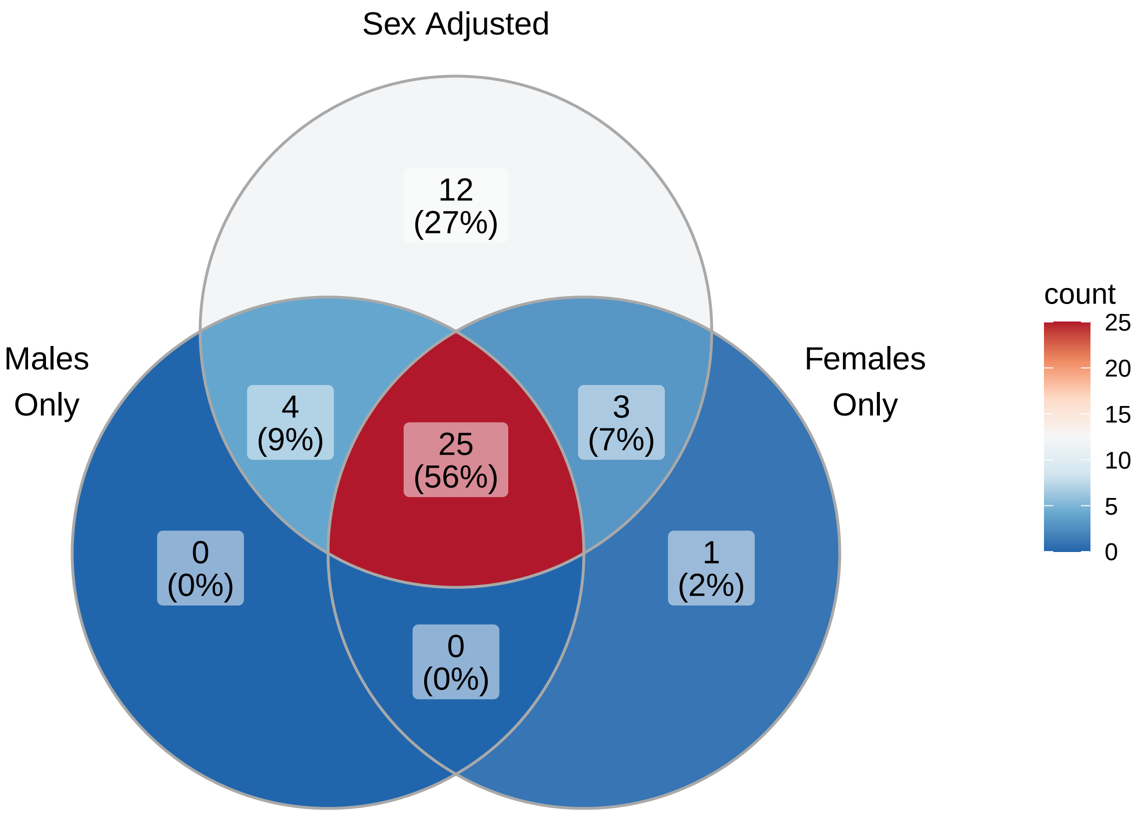

Supplementary Figure 5. Some genera are exclusively significant in sex-separated models, but are all found in the sex-adjusted significant genera. Venn diagram of the significant genera found in each of the three models. In all cases, the contrast chosen was Current/Never, adjusting for age and number of teeth. All significance thresholds were q-values < 0.05, all Benjamini-Hochberg (5% FDR) except for ALDEx2, which uses Holm correction.

Supplementary Figure 6 - Correlations log2 Fold Change values from Differential abundance and regression on daily smoking intensity

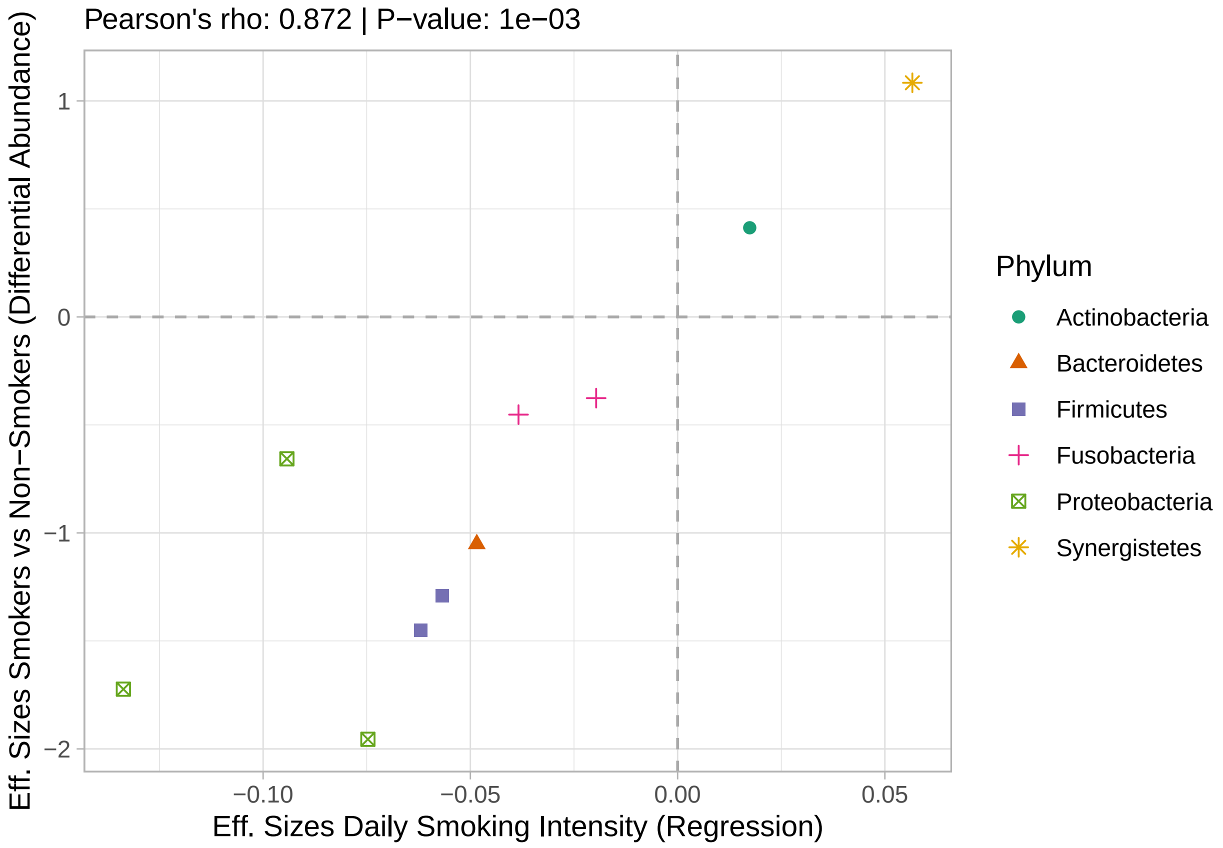

Supplementary Figure 6. Scatterplot and correlation index between effect size estimates in the differential abundance contrasting Current over Never smokers and the effect size estimates for the regression of genera abundance in relation to the daily grams of tobacco smoked daily. Both methods used DESeq2 regression correcting for age group, sex, number of teeth. However, in the comparison between Current and Never smokers, a contrast between factors was extracted (log2 Fold Change), in the second case the estimates were step-wise fold change estimates in response to increasing 5 grams smoked per day (0, 5, 10, . . . , 30 g).

Supplementary Figure 7 - Correlation of differentially abundant pathways

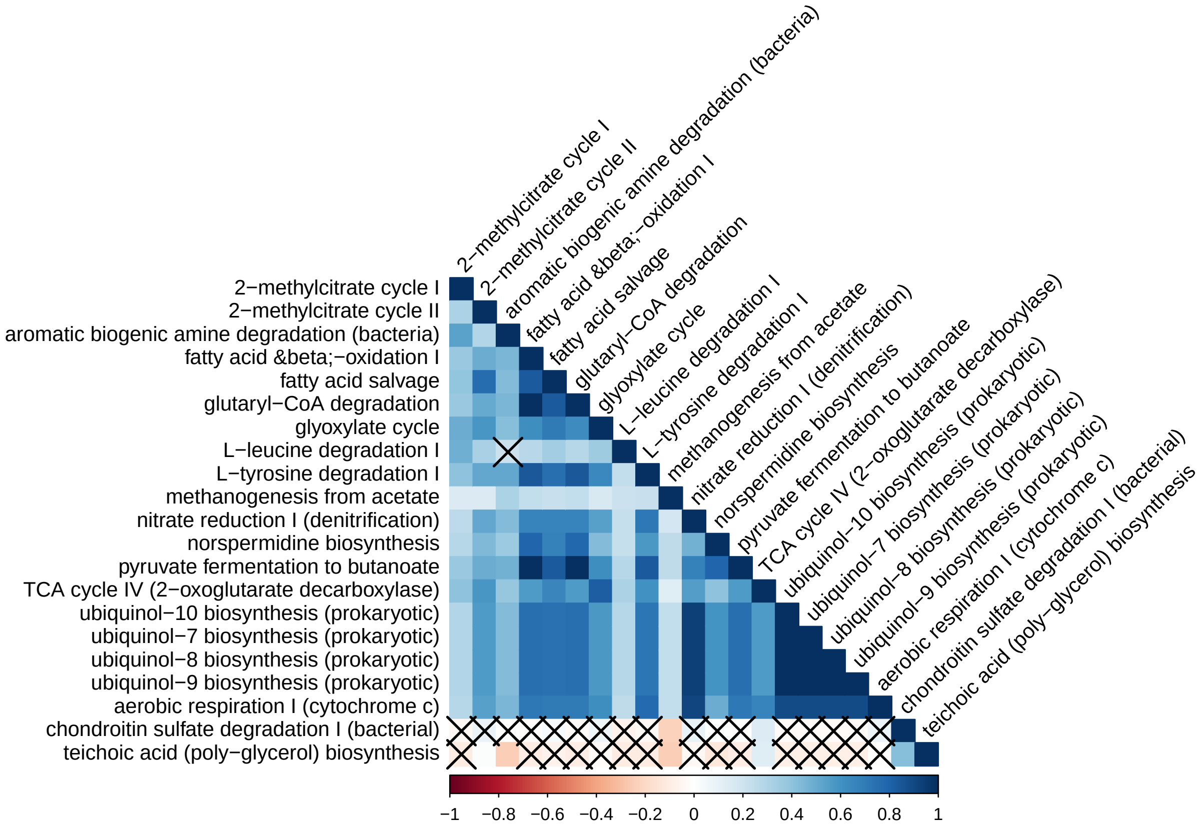

Supplementary Figure 7. Correlation among pathways that were found differentially abundant in smokers compared to never smokers. Pathways were transformed to relative abundance to account for compositionality, and a pairwise Spearman correlation matrix was calculated with the `Hmisc::rcorr` function, which additionally reports a P-value as indication of the strength of the correlation estimate. Blue circles are **positive** correlations, red circles are **negative** correlations. Larger circles mean a stronger correlation. Crossed-out slots are non-significant associations (p >= 0.01).

Supplementary Figure 8 - Gram staining relative abundances vs Smoking habits

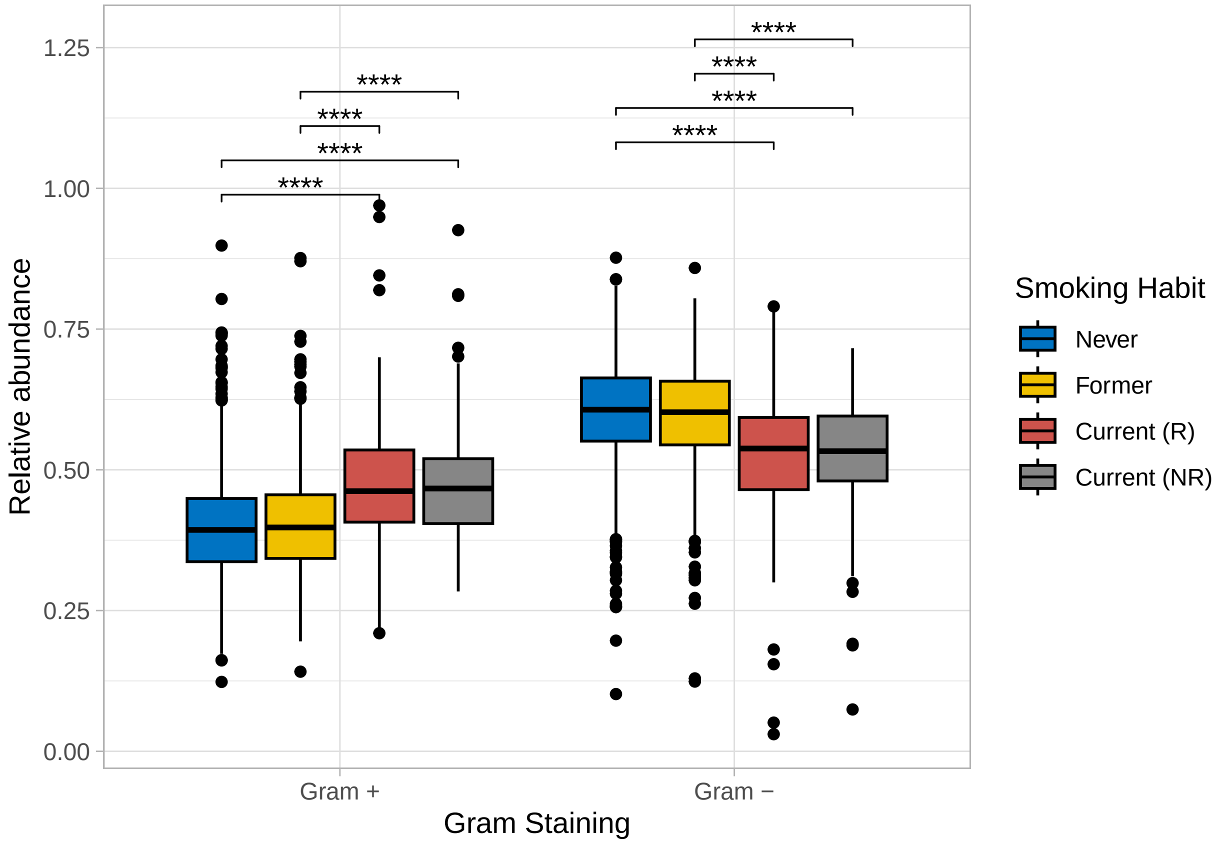

Supplementary Figure 8 - Gram Positive bacteria are more abundant in the saliva of smokers, regardless of reduction. Phyla were annotated based on a manually curated table made by G.A., since Gram staining generally differentiates at phylum level. The results were presented like the oxygen metabolism on Figure 1 (Main Text) and Supplementary Figure 6-B.

Supplementary Figure 9 - Current (NR) are indistinguishable from Current (R)

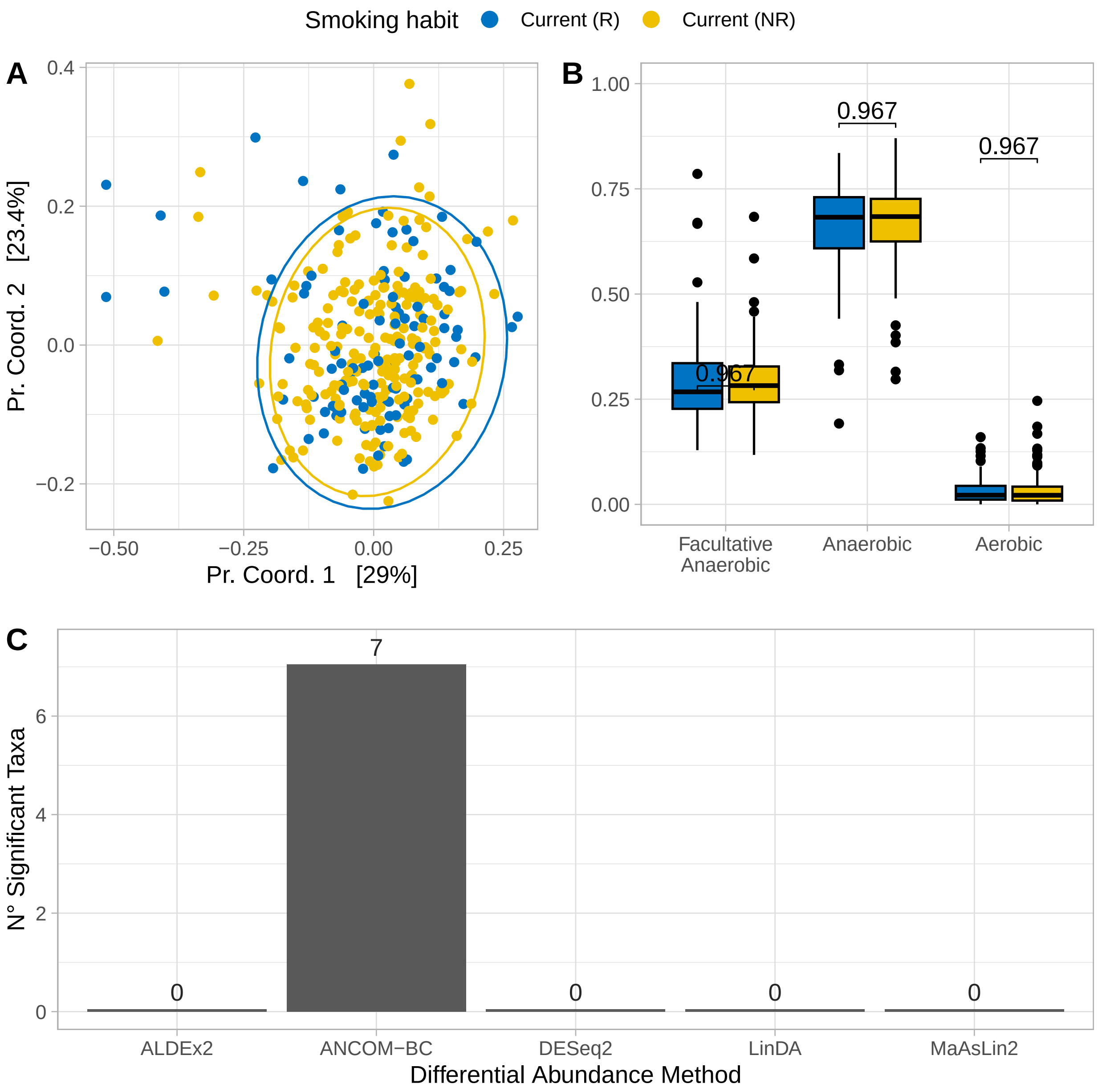

Supplementary Figure 9. Current smokers who reduced daily smoking in the past, Current (R), are indistinguishable from Current smokers who never reduced daily smoking, Current (NR) (A) Bray-Curtis distance based Principal Cordinate Analysis (PCoA) of the first 2 axes. Ellipses were drawn after the 95% confidence interval of the area covered by each smoker group. (B) Relative abundance of aerobes, anaerobes, and facultative anaerobes in Current (R) and Current (NR) smokers. Statistical significance was calculated with pairwise Wilcoxon test adjusting p-values (q-values) for a 5% FDR with the Benjamini-Hochberg method (** $q < 0.05$; *** $q < 0.001$, **** $q < 0.0001$). (C) Barplot of the number of differentially abundant taxa in the $\frac{Current(NR)}{Current(R)}$ contrast using 5 different methods (See main text for the methods). Each method considered a significant results if Benjamini-Hochberg corrected q-values were lower than 0.05, at a False Discovery Rate of 0.05, the only exception was ALDEx2, which implements only Holm's correction.

Supplementary Figure 10 - Histogram of grams per day in current smokers

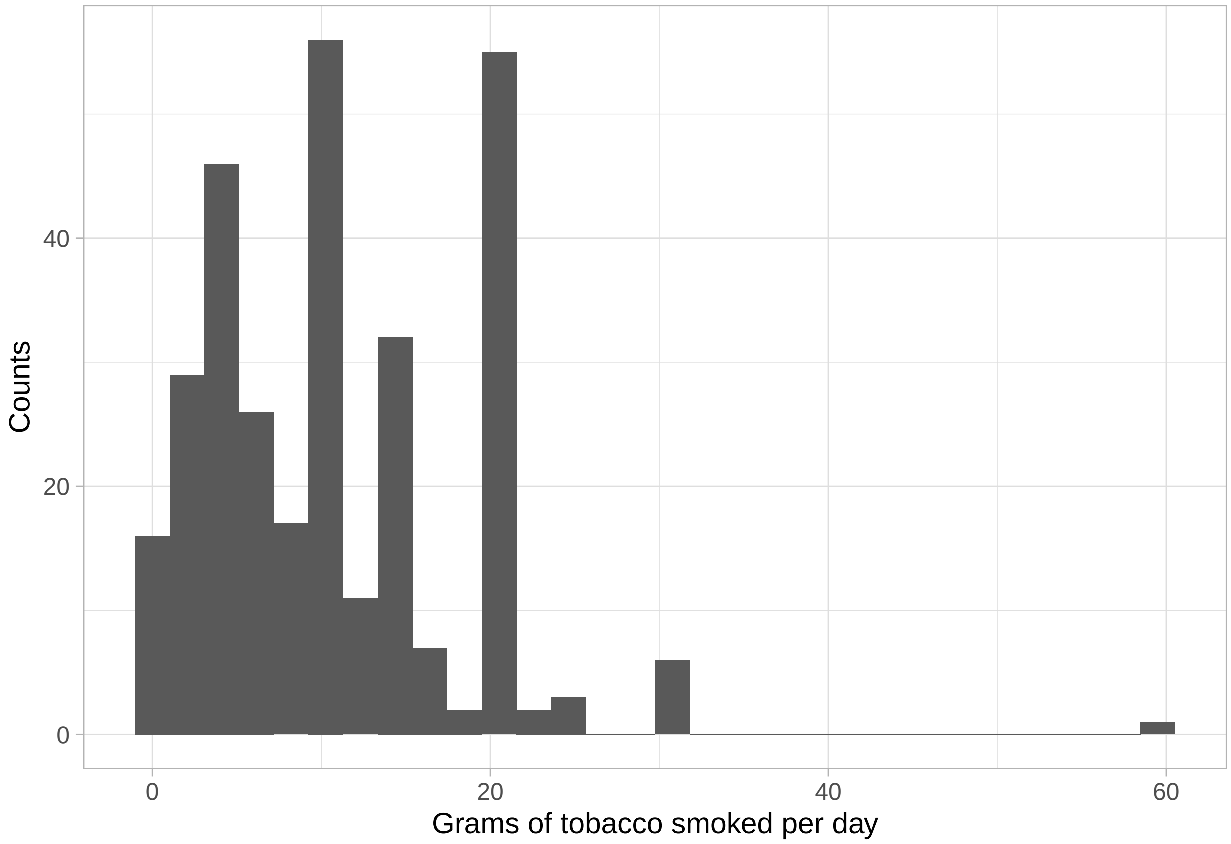

Supplementary Figure 10. Histogram of grams of tobacco smoked per day by CHRISMB smokers (N = 309) Tobacco is an integration of cigarettes, cigars, cigarillos and pipe. However, except 5 participants, all smokers were exclusively cigarette smokers. The participant who declared smoking 60 cigarettes (~ 60 grams) per day was included in this graph for completeness, but excluded from the analysis.

Supplementary Tables

Supplementary Table 1 - Literature overview, table of ethnicities and sample size of studies

Supplementary Table 1. High-level comparison of studies with comparable design Only studies investigating cigarette smoking association with the salivary microbiota and 16S amplicon sequencing technology were included.

Ethnicity	Country of cohort	Sample size	Reference
European	United States	1204	Wu et al. 2016
Japanese	Japan	2343	Takeshita et al. 2016
Unknown	United States	363	Börnigen et al. 2017
Middle-East	Saudi Arabia	330	Vallès et al. 2018
Mostly European	United States	259	Beghini et al. 2019
Low-income African American	United States	1616	Yang et al. 2019
Eastern Asia	Eastern Asia	657	Sato, Kakuta, Uchino, et al. 2020b
Eastern Asia	China	316	Jia et al. 2021
European	Denmark	746	Poulsen et al. 2022

Supplementary Table 2 - Available samples after each exclusion step

Criterion	Samples Excluded (N)	Samples after exclusion (N)
Missing Smoking Status	4	1919
Missing Number of Teeth	44	1875
Missing Antibiotic Usage Information	83	1792
Took Antibiotics in the last 3 months	191	1601

Supplementary Table 3 - No statistical differences between excluded and included participants for the study

Supplementary Table 3. Statistical differences between excluded and included participants in the study. Significance (p-value column) was calculated as Welch's t-test for continuous variables, Fisher exact test for 2x2 comparisons, and Pearson's Chi squared test for multiclass factors. Abbreviations used: BP = Blood pressure; BMI = Body Mass Index.

Characteristic	excluded, N = 322	included, N = 1,601	p-value
Smoking Habit			0.5
Never	172 (54%)	880 (55%)	
Former	90 (28%)	395 (25%)	
Current (R)	17 (5.3%)	100 (6.2%)	
Current (NR)	39 (12%)	226 (14%)	
Unknown	4	0	
Antibiotics Usage	193 (85%)	0 (0%)	<0.001
Unknown	95	0	
Systolic BP	128 (17)	126 (14)	0.12
Unknown	22	138	
Diastolic BP	79 (10)	78 (9)	0.088
Unknown	22	138	
BMI	26.0 (4.7)	25.7 (4.5)	0.3
N° Teeth			0.4
0	19 (6.8%)	89 (5.6%)	
1-9	21 (7.6%)	118 (7.4%)	
10-19	51 (18%)	239 (15%)	
20+	187 (67%)	1,155 (72%)	
Unknown	44	0	
Gum Health (self-assessed)			0.9
Excellent	11 (3.9%)	79 (5.5%)	
Very good	64 (23%)	331 (23%)	
Good	99 (35%)	502 (35%)	
Average	88 (31%)	412 (29%)	
Poor	16 (5.7%)	93 (6.5%)	
Very poor	2 (0.7%)	11 (0.8%)	
Unknown	42	173	

[1] n (%); Mean (SD)

[2] Pearson's Chi-squared test; Welch Two Sample t-test; Fisher's exact test

Supplementary Table 4 - Per taxonomic level microbiota statistics

Supplementary Table 4. CHRISMB total reads and total taxonomic ranks before and after filtering by prevalence and detection with the function 'microbiome::core'.

	**Unfiltered**	**Filtered**
Thresholds of Prevalence|Detection	-|-	0.01|10
Number of Reads	4.41e+07	4.34e+07
Kingdom	2	1
Phylum	13	11
Class	28	21
Order	46	30
Family	79	43
Genus	165	82
Species	11826	606
ASV	11860	622

Supplementary Table 5 - PERMANOVA table of variables included in the models (marginal effect)

Supplementary Table 5. Permutational (x2000) Multivariate Analysis of variance (PERMANOVA) of the marginal effect of age, sex, smoking and number of teeth in the compositional variability of the microbiota. Interindividual microbiota-based dissimilarity was calculated with the Bray-Curtis method on counts transformed to relative abundances (Total Sum Scaling, ranging from 0 to 1). The Beta dispersity (Tuckey post-hoc test significance) was P = 0.126.

**Term**	**Deg.Freedom**	**SumOfSquares**	**R2 (%)**	**F-stat**	**P-value**
Number of Teeth	3	1.9	2.4	14.4	5e-04
Sex	1	0.2	0.3	5.4	1e-03
Age Category	5	1.4	1.8	6.5	5e-04
Smoking habits (3 cat)	2	2.8	3.6	31.7	5e-04
Residuals	1589	69.5	89.7	NA	NA
Total	1600	77.5	100.0	NA	NA

Supplementary Table 6 - Linear model aerobes against smoking intensity per day

Supplementary Table 6. The relative abundance of AEROBIC bacteria decreases with increasing grams of tobacco smoked in a day. The trend is true up to 10 g/day, after which a plateau is reached.

term	estimate	std.error	statistic	p.value
Intercept	-0.007	0.012	-0.598	0.550
1/tobacco exposure intensity (g/day)	0.027	0.008	3.545	0.000
Age	0.000	0.000	1.579	0.115
Sex	0.005	0.004	1.322	0.187
1-9 teeth	0.004	0.011	0.331	0.741
10-19 teeth	0.024	0.009	2.571	0.011
20+ teeth	0.024	0.009	2.645	0.009

Supplementary Table 7 - Linear model anaerobes against smoking intensity per day

Supplementary Table 7. The relative abundance of ANAEROBIC bacteria decreases with increasing grams of tobacco smoked per day. The trend is true up to 10 g/day, after which a plateau is reached.

term	estimate	std.error	statistic	p.value
Intercept	0.611	0.035	17.244	0.000
1/tobacco exposure intensity (g/day)	-0.020	0.022	-0.941	0.347
Age	-0.001	0.000	-2.562	0.011
Sex	-0.002	0.010	-0.228	0.820
1-9 teeth	0.129	0.030	4.272	0.000
10-19 teeth	0.102	0.027	3.844	0.000
20+ teeth	0.111	0.026	4.252	0.000

Supplementary Table 8 - Linear model aerobes against years since smoking cessation

Supplementary Table 8. The relative abundance of AEROBIC bacteria increases with increasing years since smoking cessation. The trend reaches a plateau after 20 years, it is no longer statistically associated (data not shown)

term	estimate	std.error	statistic	p.value
Intercept	0.024	0.024	0.995	0.321
Years since quitting	0.001	0.001	1.944	0.053
Age	0.000	0.000	1.022	0.308
Sex	0.002	0.006	0.359	0.720
1-9 teeth	0.029	0.017	1.703	0.090
10-19 teeth	0.025	0.017	1.494	0.137
20+ teeth	0.020	0.016	1.254	0.211

Supplementary Table 9 - Linear model anaerobes against years since smoking cessation

Supplementary Table 9. The relative abundance of ANAEROBIC bacteria increases with increasing years since smoking cessation. The trend reaches a plateau after 20 years, it is no longer statistically associated (data not shown)

term	estimate	std.error	statistic	p.value
Intercept	0.024	0.024	0.995	0.321
Years since quitting	0.001	0.001	1.944	0.053
Age	0.000	0.000	1.022	0.308
Sex	0.002	0.006	0.359	0.720
1-9 teeth	0.029	0.017	1.703	0.090
10-19 teeth	0.025	0.017	1.494	0.137
20+ teeth	0.020	0.016	1.254	0.211

Supplementary Table 10 - Table 1 with Current smokers split into reducers and non-reducers

Supplementary Table 10. Demographics of CHRISMB cohort, in South Tyrol, Italy, with respect to smoking habit. Per-column percentages were also reported in brackets. Current smokers were separated into smokers who reduced daily smoking dosage some time in the past - Current (R), and those who did not reduce - Current (NR). The whole cohort is included under the "CHRISMB" column. Significance is calculated as X^2 test for categorical variables"

	Never	Former	Current (R)	Current (NR)	CHRISMB	X^2 p-value
	(N=880)	(N=395)	(N=100)	(N=226)	(N=1601)	
**Sex**						
Male	356 (40.5%)	222 (56.2%)	58 (58.0%)	115 (50.9%)	751 (46.9%)	5.1e-07
Female	524 (59.5%)	173 (43.8%)	42 (42.0%)	111 (49.1%)	850 (53.1%)	
**Age Category (years)**						
18-30	238 (27.0%)	41 (10.4%)	38 (38.0%)	92 (40.7%)	409 (25.5%)	2.2e-17
31-40	139 (15.8%)	73 (18.5%)	18 (18.0%)	39 (17.3%)	269 (16.8%)	
41-50	196 (22.3%)	75 (19.0%)	23 (23.0%)	41 (18.1%)	335 (20.9%)	
51-60	144 (16.4%)	112 (28.4%)	12 (12.0%)	39 (17.3%)	307 (19.2%)	
61-70	93 (10.6%)	57 (14.4%)	8 (8.0%)	15 (6.6%)	173 (10.8%)	
71+	70 (8.0%)	37 (9.4%)	1 (1.0%)	0 (0%)	108 (6.7%)	
**N° Teeth (self-reported)**						
0	50 (5.7%)	23 (5.8%)	7 (7.0%)	9 (4.0%)	89 (5.6%)	0.11
1-9	57 (6.5%)	41 (10.4%)	4 (4.0%)	16 (7.1%)	118 (7.4%)	
10-19	117 (13.3%)	74 (18.7%)	13 (13.0%)	35 (15.5%)	239 (14.9%)	
20+	656 (74.5%)	257 (65.1%)	76 (76.0%)	166 (73.5%)	1155 (72.1%)	
**Gums Health (self-reported)**						
Excellent	45 (5.1%)	18 (4.6%)	4 (4.0%)	12 (5.3%)	79 (4.9%)	0.44
Very good	188 (21.4%)	79 (20.0%)	20 (20.0%)	44 (19.5%)	331 (20.7%)	
Good	291 (33.1%)	124 (31.4%)	30 (30.0%)	57 (25.2%)	502 (31.4%)	
Average	229 (26.0%)	84 (21.3%)	27 (27.0%)	72 (31.9%)	412 (25.7%)	
Poor	47 (5.3%)	24 (6.1%)	9 (9.0%)	13 (5.8%)	93 (5.8%)	
Very poor	6 (0.7%)	2 (0.5%)	3 (3.0%)	0 (0%)	11 (0.7%)	
Missing	74 (8.4%)	64 (16.2%)	7 (7.0%)	28 (12.4%)	173 (10.8%)	

Supplementary Table 11 - eHOMD database expansion, list of genomes accession numbers per species

Supplementary Table 11. Genome accession numbers (NCBI codes) for each species added to the eHOMD reference database. The 16S rRNA genes of each genome were added to the locally downloaded extended Human Oral Microbiome Database (eHOMD) in order to maximize the likelihood of species level taxonomic assignment.

Species	NCBI Reference Sequences
Acinetobacter haemolyticus	NR_026207.1, NR_117622.1, NR_119359.1
Actinomyces naeslundii	NR_037033.1, NR_113326.1, NR_118900.1
Actinomyces oris	NR_117358.1, NR_113060.1, NR_104896.1
Cardiobacterium hominis	NR_025934.1
Corynebacterium matruchotii	NR_119179.1, NR_026216.1
Lactobacillus casei	NR_118976.1, NR_115322.1, NR_113333.1, NR_115534.1, NR_041893.1
Lactobacillus fermentum	NR_118978.1, NR_113335.1, NR_104927.1
Lactobacillus rhamnosus	NR_113332.1, NR_043408.1
Neisseria flavescens	NR_025892.1
Neisseria sicca	NR_121688.1
Neisseria subflava	NR_041989.1
Prevotella melaninogenica	NR_042843.1, NR_102895.1, NR_113113.1, NR_042843.1, NR_102895.1
Prevotella pleuritidis	NR_041541.1
Prevotella salivae	NR_024816.1, NR_024815.1
Rothia mucilaginosa	NR_044873.1
Streptococcus mitis	NR_115560.1, NR_116207.1, NR_115732.1, NR_115240.1, NR_028664.1
Streptococcus mutans	NR_118933.1, NR_115733.1, NR_116208.1, NR_114726.1, NR_113641.1, NR_042772.1
Streptococcus oralis	NR_115734.1, NR_116210.1, NR_114413.1, NR_042927.1, NR_118234.1, NR_117719.1
Streptococcus sobrinus	NR_118935.1, NR_114727.1, NR_042773.1
