# Supplementary material for: Smoking and salivary microbiota: a cross-sectional analysis of an Italian alpine population
Source: Sci Rep. 2023 Nov 2;13:18904. doi: 10.1038/s41598-023-42474-7 (PMC10622503; doi:10.1038/s41598-023-42474-7)
Supplement: Supplementary file 2 — Supplementary Information 2. [file 41598_2023_42474_MOESM2_ESM.pdf]

Smoking and salivary microbiota: a cross-sectional analysis of an Italian alpine population

Controls Analysis

Giacomo Antonello, Freida Blostein, Deesha Bhaumik, Elyse Davis, Martin Gögele, Roberto Melotti, Peter Pramstaller, Cristian Pattaro, Nicola Segata, Betsy Foxman, Christian Fuchsberger

2023-08-20

Contents

PCoA (Beta diversity) split by controls **2**

Zymo Mock communities composition (Positive Controls) **3**

Heatmap by ASV 3

Barplot by ASV 4

How much variability does the Batch explain? 5

Table 1: Number of samples per control type (columns) in each sequencing batch (rows)

	DNA extraction (Neg)	Water (Neg)	Zymo Mock (Pos)
B. 1	3	3	3
B. 2	4	4	4
B. 3	4	4	4
B. 4	4	4	4
B. 5	4	4	4
B. 6	4	11	4
B. 7	4	4	4
B. pilot_5	1	0	1
B. pilot_6	1	0	1
B. pilot_7	1	0	1

PCoA (Beta diversity) split by controls

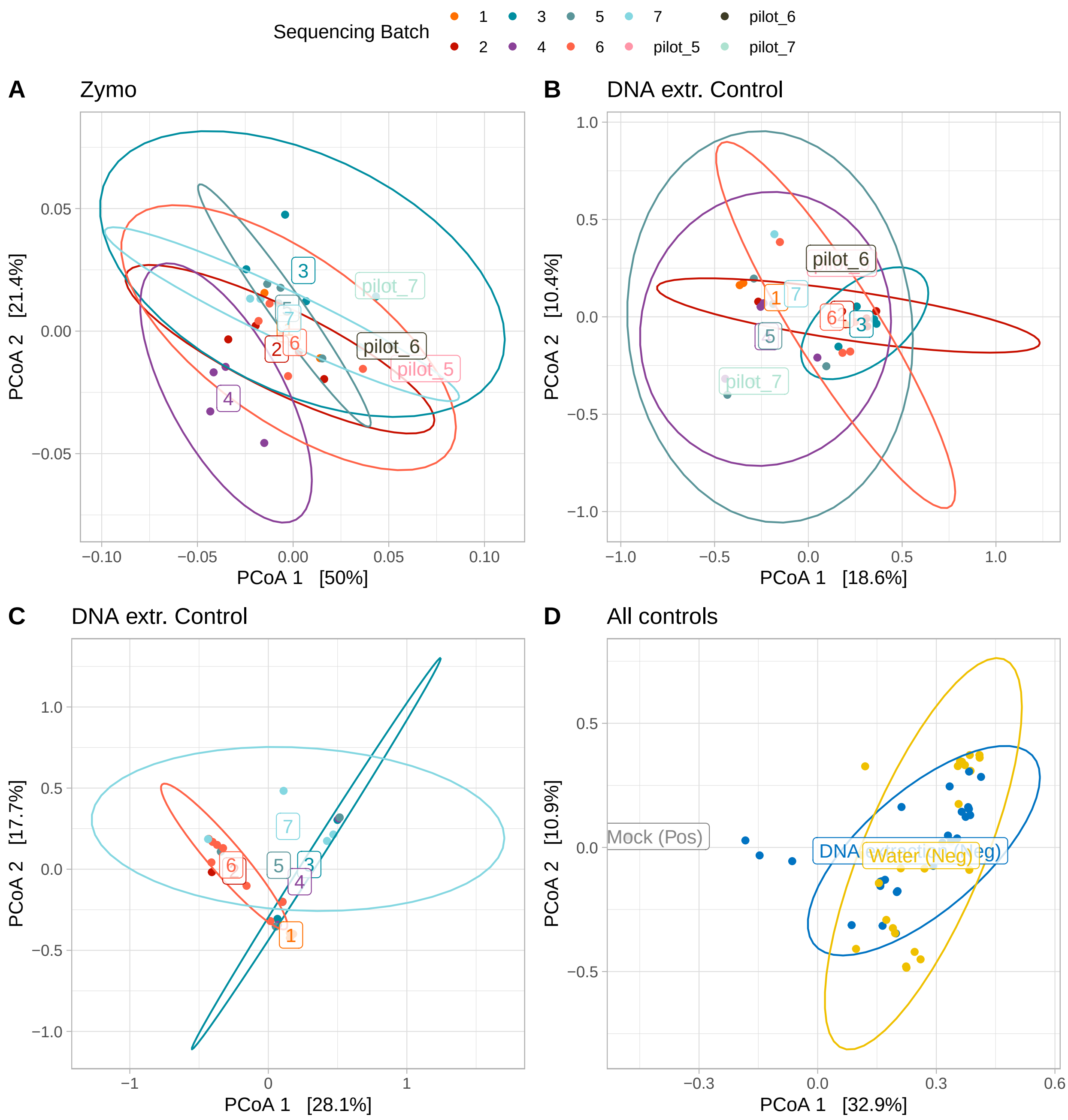

Figure 1. Bray-Curtis distance based Principal Coordinate Analysis plots of Controls (A) Zymo Mock community positive control. (B) Water extraction control. (C) DNA Extraction Control. The **legend on top** of the table refers to panels **A-C only** (D) Same visualization, but all control types were included, which induced a clear separation between negative and Zymo positive controls. The Bray-Curtis distance was calculated on the whole data set at first, then subset every time to keep only available samples. This was done to induce further separation based also on the taxa unique to the Zymo and the negative controls.

Zymo Mock communities composition (Positive Controls)

Heatmap by ASV

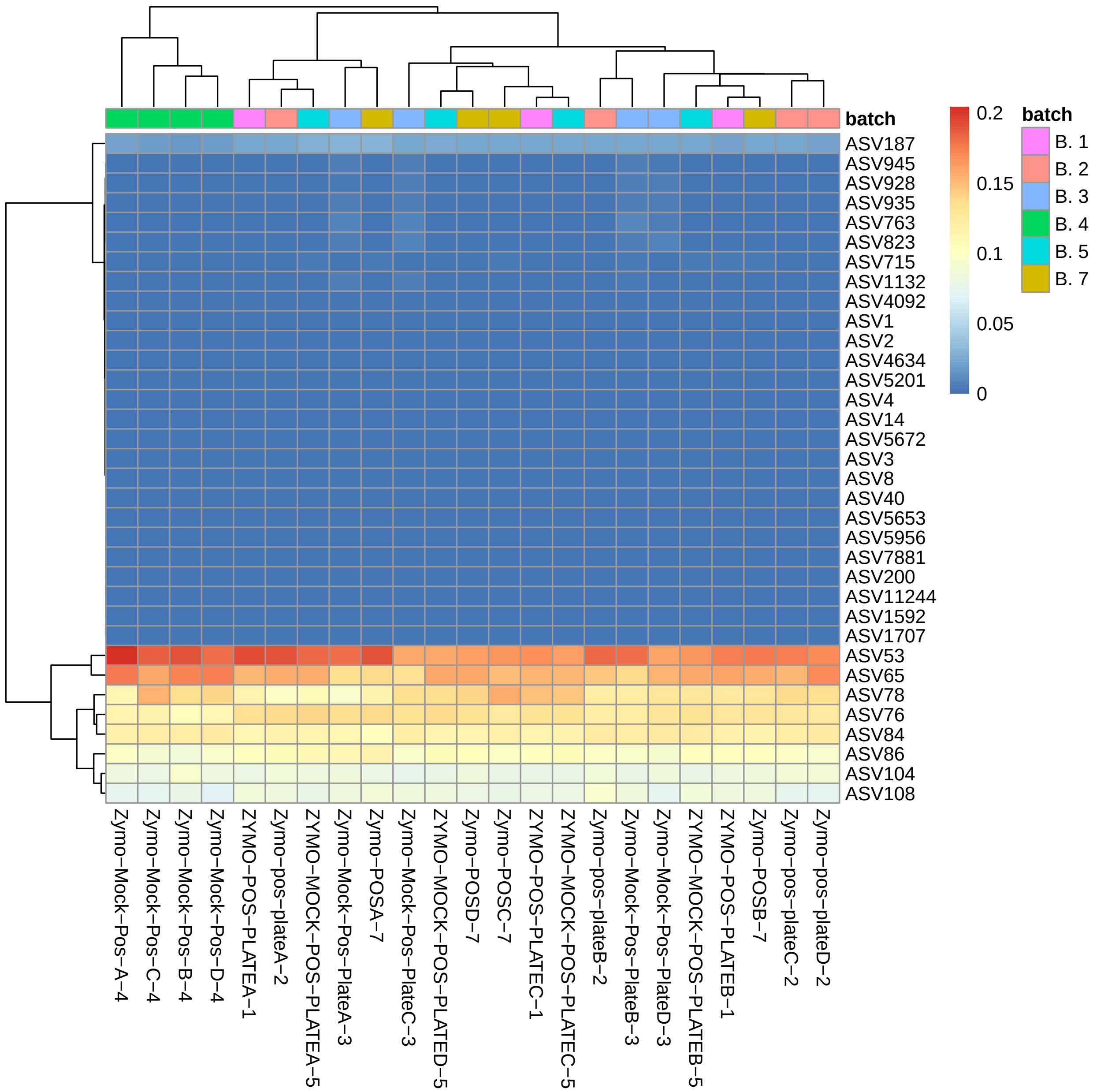

Figure 2. Heatmap of the relative abundance taxonomic composition at ASV level of Zymo Mock communities No scaling except the relative abundance transformation was applied to the ASVs. Clustering was performed on both rows and columns. Columns were annotated by sequencing batch.

Barplot by ASV

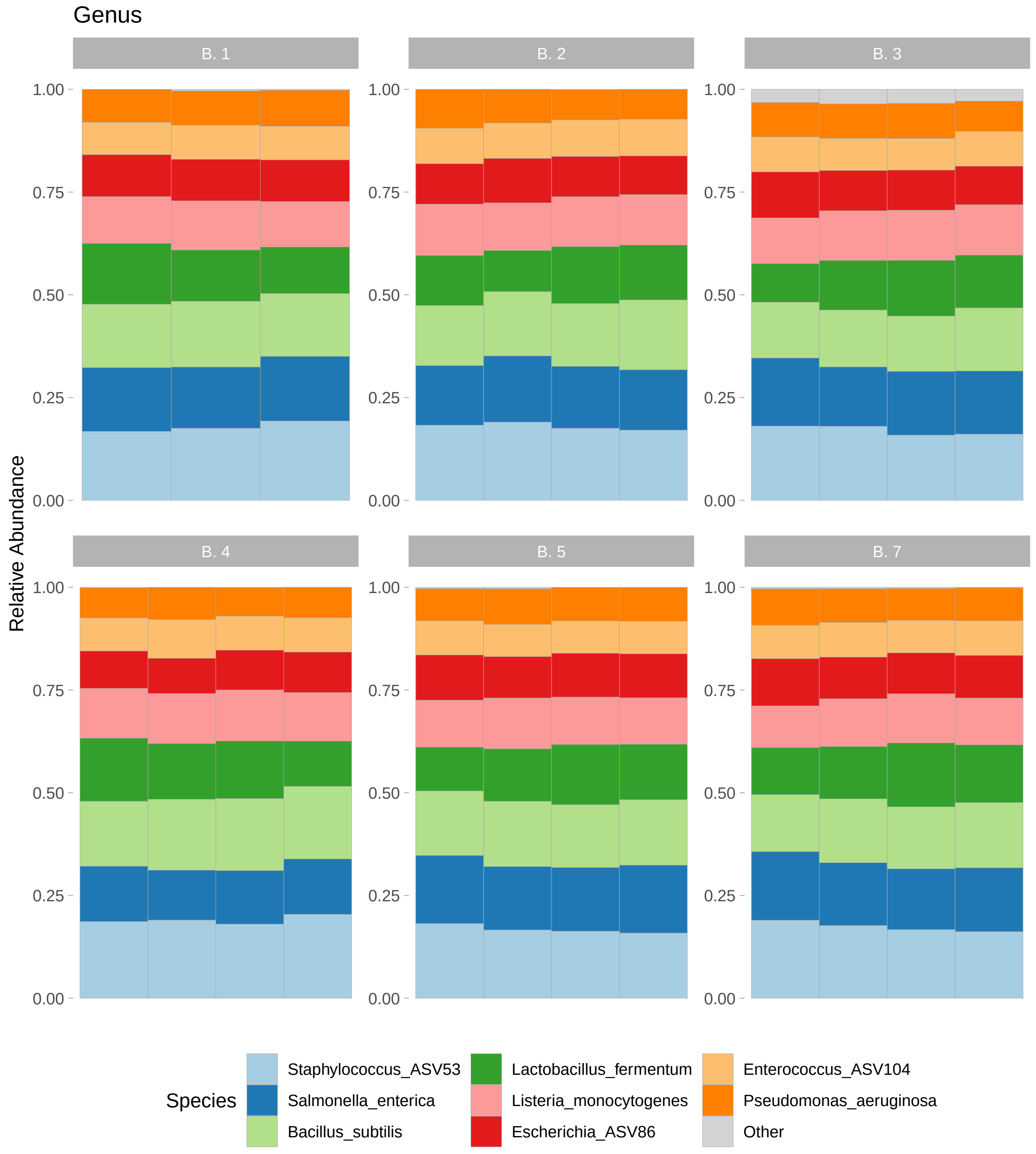

Figure 3. Relative abundance of Species found in each Zymo Mock sample viewed as stacked barplots Samples were separated (faceted) by sequencing batch. The legend is ordered by columns based on decreasing taxon abundance across all mock samples.

How much variability does the Batch explain?

Table 2: PERMUTEST (Tukey post-hoc) with default settings on the beta-dispersity in relation to batch

	Df	Sum Sq	Mean Sq	F	N.Perm	Pr(>F)
Groups	5	0.000	0	0.225	999	0.943
Residuals	17	0.003	0	NA	NA	NA

Table 3: Bray-Curtis distance-based PERMANOVA with standard settings to view the variability partitioning and significance in relation to sequencing batch and DNA extraction plate for Zymo Mock communities (Positive Controls)

term	df	SumOfSqs	R2	statistic	p.value
plate	3	0.012	0.402	20.254	0.001
batch	5	0.015	0.506	15.309	0.001
Residual	14	0.003	0.093	NA	NA
Total	22	0.030	1.000	NA	NA
